# The StUBC18-StPUB40 pair negatively regulate drought stress tolerance and influences tuber yield in potato

**DOI:** 10.1093/hr/uhaf145

**Published:** 2025-06-10

**Authors:** Weigang Liu, Xun Tang, Rui Ma, Jiangwei Yang, Xue Fu, Huanhuan Zhang, Shigui Li, Ning Zhang, Huaijun Si

**Affiliations:** State Key Laboratory of Aridland Crop Science, Gansu Agricultural University, Yingmencun No.1, Anning District, Lanzhou 730070, China; College of Agronomy, Gansu Agricultural University, Yingmencun No.1, Anning District, Lanzhou 730070, China; State Key Laboratory of Aridland Crop Science, Gansu Agricultural University, Yingmencun No.1, Anning District, Lanzhou 730070, China; College of Life Science and Technology, Gansu Agricultural University, Yingmencun No.1, Anning District, Lanzhou 730070, China; State Key Laboratory of Aridland Crop Science, Gansu Agricultural University, Yingmencun No.1, Anning District, Lanzhou 730070, China; College of Agronomy, Gansu Agricultural University, Yingmencun No.1, Anning District, Lanzhou 730070, China; State Key Laboratory of Aridland Crop Science, Gansu Agricultural University, Yingmencun No.1, Anning District, Lanzhou 730070, China; College of Life Science and Technology, Gansu Agricultural University, Yingmencun No.1, Anning District, Lanzhou 730070, China; State Key Laboratory of Aridland Crop Science, Gansu Agricultural University, Yingmencun No.1, Anning District, Lanzhou 730070, China; College of Agronomy, Gansu Agricultural University, Yingmencun No.1, Anning District, Lanzhou 730070, China; State Key Laboratory of Aridland Crop Science, Gansu Agricultural University, Yingmencun No.1, Anning District, Lanzhou 730070, China; College of Agronomy, Gansu Agricultural University, Yingmencun No.1, Anning District, Lanzhou 730070, China; College of Life Science and Technology, Gansu Agricultural University, Yingmencun No.1, Anning District, Lanzhou 730070, China; State Key Laboratory of Aridland Crop Science, Gansu Agricultural University, Yingmencun No.1, Anning District, Lanzhou 730070, China; College of Life Science and Technology, Gansu Agricultural University, Yingmencun No.1, Anning District, Lanzhou 730070, China; State Key Laboratory of Aridland Crop Science, Gansu Agricultural University, Yingmencun No.1, Anning District, Lanzhou 730070, China; College of Life Science and Technology, Gansu Agricultural University, Yingmencun No.1, Anning District, Lanzhou 730070, China

## Abstract

The ubiquitin–proteasome system (UPS) is important for protein post-translational modification in plants. E2 (ubiquitin-conjugating enzyme) and E3 (ubiquitin ligases enzyme), key enzymes of UPS, play crucial roles in all aspects of plant development, growth, and environmental stresses. Despite extensive knowledge of UPS roles in crop growth and development, E2-E3 pair functions in potato tuber development and stress responses remain understudied. Here, we describe the role of StUBC18 (a potato E2) in drought stress tolerance. It is determined that *StUBC18* (E2)-StPUB40 (E3) pair plays important roles in drought stress tolerance and potato tuber yield. *StUBC18* and *StPUB40* expression was downregulated under various stresses (drought, salt, polyethylene glycol, and H_2_O_2_). Overexpression of *StUBC18* and *StPUB40* in potatoes decreased drought stress tolerance, while interfering with the expression of *StUBC18* and *StPUB40* increased drought stress tolerance, respectively. The protein interaction test demonstrated that StUBC18 interacts with StPUB40 in the plant cell. Co-overexpression of StUBC18-StPUB40 in potato enhanced reactive oxygen species (ROS) accumulation and induced pleiotropic changes, reducing drought tolerance. Our findings revealed how the StUBC18-StPUB40 pair regulates potato drought stress tolerance by altering leaf anatomy (palisade and spongy tissue thickness) and influences tuber yield.

## Introduction

Plants, being sessile and autotrophic organisms, are exposed to environmental challenges throughout their life cycle, including biological stress (herbivores, pests, pathogens) and abiotic stress (e.g. drought, high salt, temperature fluctuations, metals, nutrients, air pollution, and photooxidation stress) [1, 2]. Unfavorable growth conditions increase free radical levels, which cause protein denaturation and damage to the plant. Plants have evolved multiple sophisticated control systems to mitigate the damaging effects and achieve homeostasis [3]. In multiple coping strategies, the ubiquitin–proteasome system (UPS) maintains the relative balance and stability of the intracellular environment by modulating and controlling protein abundance, activity, and stability. Classically, target proteins are selectively tagged by ubiquitin and degraded by 26S proteasome if they are redundant or abnormal and misfold [4]. However, some studies have reported that the conjugation of ubiquitin to other proteins could change the substrate’s metabolic stability and co-regulator for nonproteolytic functions [5].

Ubiquitination is a key post-translational modification (PTM) and deeply involved in the regulation of all aspects of plant physiology [6], such as biotic and abiotic stress response, hormone signaling transduction, growth, and development [7]. Ubiquitin is covalently linked to substrate proteins via multilayered, reversible enzymatic reactions [8]. Three major enzymes catalyze the ubiquitination of target proteins: E1 (Ubiquitin-activating enzyme), E2 (Ubiquitin-conjugating enzyme), and E3 (Ubiquitin ligase enzyme) [6].

Monoubiquitination and multi-monoubiquitination can change the target protein’s cellular localization, interaction, activity, and function, whereas the ubiquitination hydrolysis of proteins usually depends on polyubiquitination [9]. During the assembly process of the ubiquitin chains, ubiquitin can subsequently be decorated by further ubiquitin at two or more potential post-translational modification sites (Lys6, Lys11, Lys27, Lys29, Lys33, Lys48, and Lys63) or the Met1(N-terminal methionine) [5], which form various homo- and heterotypic linkage types of polyUb chains. It has been reported that K11 polyUb chains and K48 polyUb chains are the most potent signals for degradation [10]; K63 polyUb chains are associated with hormonal responses and development, nutritional responses, autophagy, biotic interactions, and DNA repair [6]. Less is known about the roles of noncanonical protein ubiquitination beyond K48, K63, and K11 [11].

E2 is an integral part of UPS, which has a dual role of Ub delivery and Ub linkage assembly preference and determines the linkage specificity of the polyubiquitin chains [12, 13]. The E2s possess a core conserved UBC domain containing a Cys active site to form thioester linkages with E1s and E3s. E3s, as a key hub to integrate E2 and substrate, are responsible for recruiting E2 and selecting the substrate protein to be ubiquitinated [14].

According to the current understanding, E3s are not independent of E2s in the ubiquitination of target proteins, instead working as an E2-E3 pair. There has been much research on E3s and E2s independent regulatory mechanisms, but few studies have addressed the E2-E3 pair. UBC27 interacts with AIRP3 (RING E3 ligase AIRP3) to form an E2-E3 pair, UBC27-AIRP3, which mediates ubiquitination and degradation of ABI1 (protein phosphatase 2Cs, PP2Cs) to modulate abscisic acid (ABA) signaling in Arabidopsis [15]. UBC26 coupled with RFA4 (RING between RING fingers (RBR)-type RSL1/RFA family) to form UBC26-ABA receptor (PYL/PYL4)-RFA4 complexes in nuclear speckles, which negatively modulate ABA receptor levels and signaling [16]. The UBC32-Rma1 (RING-type E3 ligase) complex can integrate with Ser280/283-phosphorylated of PIP2;1, facilitating ubiquitination of PIP2 at Lys276 and mediating its degradation, thereby increasing plant drought tolerance [17]. Recent studies have revealed that GhUBC2L interacts with U-box E3s GhPUB8 synergistically with mediating histone monoubiquitination to modulate organ size in cotton (*Gossypium hirsutum*) [18]. In addition, the E2 enzymes are also supervised by the UPS system; e.g. UBC32 related to ERAD (ER-associated protein degradation) can be ubiquitinated by E3 ligase HRD1 to regulate ERAD activity [19].

In previous studies, several E2 enzymes were involved in plant development and abiotic stress responses [18, 20]. Here, we characterized the function of StUBC18 in drought stress tolerance. During our quest to discover the mechanisms by which UBC18 responds to drought stress in potato, we found that StUBC18 can interact with StPUB40 to form an E2-E3 pair, StUBC18-StPUB40. Here, we deciphered the molecular mechanism by which the StUBC18-StPUB40 pair responds to drought stress and influences tuber yield in potato.

## Results

### Isolation and characterization of StUBC18 from potato

**Figure 1:**
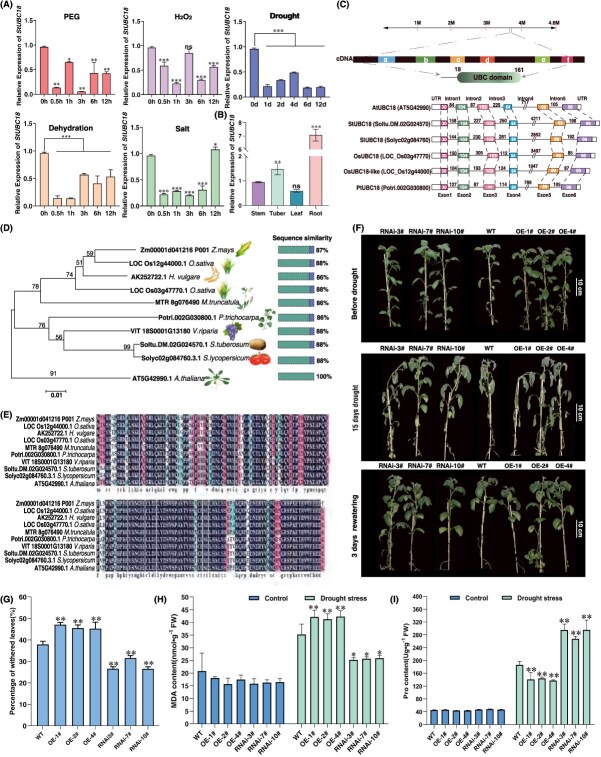
Characterization analysis of *StUBC18*. (A) The expression of *StUBC18* under different stress. (B) The organ-specific expression of the *StUBC18* in potato. (C) Structure of full-length *StUBC18* gene, and phylogenetic analysis of UBC18 from potato and nine other species. (D) The phylogenetic tree analysis of UBC18 from potato and nine other species. (E) Multiple alignments of an amino acid sequence of UBC18 from potato and nine other species. (F) Phenotype of transgenic potato plants from restriction watering 15 days to 3 days rewatering. (G) The change in percentage of withered leaves of transgenic potato plants after 15 days drought stress. (H) The change of transgenic potato plants MDA content under drought stress. (I) The change of transgenic potato plants Pro content under drought stress. Asterisk marks above bars indicate significant differences (The data were determined by ANOVA followed by Tukey *post hoc* test, *n* = 3, ^*^P < 0.05, ^**^P < 0.01, ^***^P < 0.001).

In our previous study, 57 putative StUBCs were identified in potatoes [21]. In this study, to evaluate the probable role of *StUBC18* (Soltu.DM.02G024570.1) in potato growth and development, we detected *StUBC18* transcript levels during several treatments and tissue specificity by quantitative reverse transcription-polymerase chain reaction (qRT-PCR). The gene expression profiles of the various treatments showed altered *StUBC18* gene expression. *StUBC18* was markedly downregulated under H_2_O_2_/drought/salt/PEG/dehydration stress (Fig. 1A). Thus, *StUBC18* functions in response to abiotic stress. The qRT-PCR analysis demonstrated that *StUBC18* was expressed in root, leaf stem, and tuber (Fig. 1B). Moreover, the relative expression level of the root was the highest, and the leaf was the lowest. *StUBC18* is functionally implicated in growth and development. To elucidate the functionality and mechanism of *StUBC18* responding to drought stresses in potatoes, we cloned *StUBC18* from potted potatoes for further research. The *StUBC18* gene is composed of six exons and five introns (Fig. 1C), and the length of the open reading frame (ORF) was 486 bp, encoding a 161-amino acid protein with a UBC domain (predicted by SMART, http://smart.embl-heidelberg.de/) (class I) [13]. The phylogenetic tree analysis showed that StUBC18 was most closely related to SlUBC18 (tomato, Soly02g084760.3.1) (Fig. 1D). The sequence homology analyses revealed that the sequence identity of UBC18 between potato and tomato is 99.38%, with a single difference at the 50th amino acid (N-I) (Fig. 1E).

**Figure 2:**
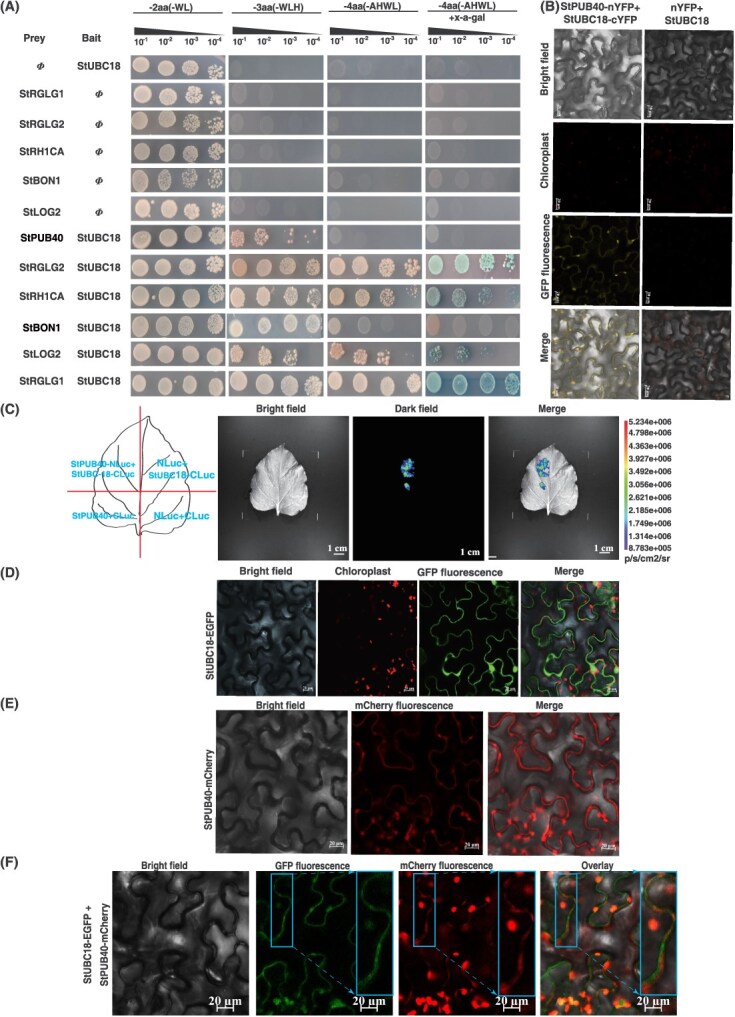
StUBC18 interacts with multiple E3s and subcellular localization. (A) Y2H assays of StUBC18 and multiple E3s. (B) The interaction between StUBC18 and StPUB40 in BiFC assay. Scale bar = 20 μm. (C) The firefly luciferase complementation imaging assay of StUBC18 and StPUB40 interactions. The color scale reflects LUC activity. (D) Subcellular localization of *StUBC18* in epidermal cells. Scale bar = 20 μm. (E) Subcellular localization of *StPUB40* in epidermal cells. Scale bar = 20 μm. (F) Colocalization of *StUBC18* and *StPUB40* in epidermal cells. Scale bar = 20 μm.

### StUBC18 negatively regulates potato drought tolerance

Arabidopsis UBC18 regulates drought/salt tolerance by ubiquitinating ERF1 to control its stability [22], it is not unreasonable to hypothesize that its homolog in potato could also be implicated in drought tolerance. To test this hypothesis, we performed a yeast two-hybrid (Y2H) analysis to identify whether StUBC18 interacted with StERF1 in potatoes. We failed to detect the interaction between StUBC18 and StERF1 in Y2H assay (Fig. S1A). Similarly, the SLC (Split Luciferase Complementation) and BiFC (Bimolecular Fluorescence Complementation) assay did not yield fluorescence signal in tobacco leaves, which indicated that StERF1 is not an interactor of StUBC18 in plant cells (Fig. S1B and C). This finding indicated that StUBC18 might be involved in response to drought stress through other avenues in potatoes. To further test this hypothesis and explore the roles and physiological functions of *StUBC18* in response to drought stress, the *StUBC18* gene was transformed into potato cultivar ‘Atlantic’ to produce overexpression lines (OE) (Fig. S2A–O). RNAi-StUBC18 plants were also generated in the ‘Atlantic’ background. All the transgenic lines were screened for resistance to kanamycin (Fig. S2O) and confirmed by RT-PCR. Thirty-seven overexpression explants were assessed. Among them, three positive transgenic lines (OE-1#, OE-2#, OE-4#) were identified as they had markedly increased expression levels compared to the control (Fig. S2P). After screening 41 RNA interference explants and analyzing the interfering effect of the miRNA, three independent transgenic lines (RNAi-3#, RNAi-7#, RNAi-10#) transcription levels were decreased significantly and selected for further study (Fig. S2P).

To substantiate whether *StUBC18* contributes to abiotic stress tolerance, the OE-StUBC18 plants (OE-1#, OE-2# and OE-4#) and RNAi plants (RNAi-3#, RNAi-7#, RNAi-10#) were subjected to water deficit stress in soil. None of the plants displayed significant differences in growth under normal watering conditions. However, OE plants showed more susceptibility to drought stress compared with wild type (WT) plants (Fig. 1F). Instead, RNAi plants could enhance drought tolerance compared with WT plants. Moreover, all the plants exhibited wilting phenotype in soil treated with water deficit stress, with the OE plants displaying a more prominent phenotype of wilting. In addition, the percentage of withered leaves of OE plants was significantly more than RNAi plants (Fig. 1G). The Malondialdehyde (MDA) content in OE plants was markedly higher than that in RNAi plants under drought stress (Fig. 1H). Proline (Pro) content in OE plants, meanwhile, was significantly lower than that of RNAi plants (Fig. 1I). These results showed that *StUBC18* negatively regulated potato drought stress tolerance.

### StUBC18 physically interacts with StPUB40

Through preliminary experiments, 72 positive clones were identified, including several candidate E3s that are U-box and RING-type E3 ligases (Table S2). All positive candidate clones were analyzed using Gene Ontology (GO) and Kyoto Encyclopedia of Genes and Genomes (KEGG) enrichment by Tbtools [23]. The results were analyzed using the OmicShare tools (a free online data analysis) (https://www.omicshare.com/tools). GO analysis showed that many StUBC18 partners were associated with ubiquitination and response to stimulus (Fig. S3A). In addition, KEGG analysis implicated candidates in metabolism, genetic information, and environmental processing (Fig. S3B). To confirm the interaction between StUBC18 and E3s under stringent conditions, we selected the following candidate E3s that were studied extensively to investigate by Y2H assays: StBON2, StLOG2, StRGLG1, StRGLG2, StRH1CA, and StPUB40. The full-length coding regions of these six E3s were constructed into the pGADT7 plasmid vector as prey, and StUBC18 was cloned into the pGBKT7 plasmid vector as bait, which were cotransformed into yeast cells. The results revealed that co-expression of StUBC18 and StBON2, StLOG2, StRGLG1, StRGLG2, StRH1CA, and StPUB40 conferred on yeast host strain the growth ability to grow normally in the SD (−T/−L/–H), SD (−T/−L/–H/−A) selection medium while empty vectors combination did not (Fig. 2A). The proteins with strength of interplay from strongest to weakest were StRGLG2, StRH1CA, StRGLG1, StLOG2, StBON2, and StPUB40 (Fig. 2A). In addition, the β-galactosidase quantitative assays provide evidence to corroborate the result (Fig. S3C). These findings indicated that StUBC18 is an interacting partner of these E3s. Based on previous research in Arabidopsis, we confirmed multiple E3s and their target proteins, which were involved in regulating multiple signal pathways, such as ABA, Gibberellic Acid (GA), Ethylene (ET), and auxin (Fig. S3D). Our previous result showed that the U-box ubiquitin ligase enhanced ubiquitination modification under PEG-induced drought stress [24, 25]. Therefore, we were predominantly interested in studying the role of StPUB40 in potatoes during drought stress, and focused subsequent work in this direction.

To further detect their interactions in the plant cell, BiFC and SLC experiments were employed to test the physical interaction between StUBC18 and StPUB40. The different combinations were transiently expressed in *Nicotiana benthamiana* leaves. Through confocal fluorescence microscopy, we observed YFP fluorescence signals on the cell membrane, while all of the control combinations failed to form fluorescence signal (Fig. 2B). Interestingly, there is a reconstituted luciferase activity signal in only the StPUB40-NLUC+StUBC18-CLUC combination when co-expressed with four different combinations, but not in the controls (Fig. 2C). These results indicate that StUBC18 can interact with StPUB40 in the plant cell. These results suggest that StUBC18 interacts with StPUB40 on the plant cell membrane.

Subcellular localization analysis was performed to test StUBC18 and *StPUB40* localization. We constructed two vectors driven by the 35S promoter: StUBC18-EGFP (green fluorescent protein) and StPUB40-mCherry (red fluorescent protein). Samples infiltrated by StUBC18-EGFP yielded an EGFP fluorescence signal predominantly in the nucleus and on the cell membrane (Fig. 2D). StPUB40-mCherry localized to cell membrane (Fig. 2E). Furthermore, colocalization of StBZR1-EGFP and StPUB40-mCherry generated fluorescence signals predominantly on the cell membrane (Fig. 2F), proving that StUBC18 may interact with StPUB40 on the cell membrane to regulate target protein.

StPUB40 consists of a U-box domain (22–88 aa), an unknown domain (29–210 aa), and four ARM motifs (ARM1, ARM2, ARM3, ARM4) (Fig. S4A). It has been suggested that the U-box domain confers E3 activities when conjugated with specific E2, whereas ARM motifs are responsible for associating with specific target proteins. In this study, the Y2H, SLC, and BiFC results showed that StUBC18 interacts with StPUB40. To examine the interaction domain, StPUB40 was split into different fragments by a series of deletions and truncation (Fig. S4B). Notably, one-to-one Y2H showed that the U-box domain and ARM motifs could interact with StUBC18 but much more weakly than full-length StPUB40 (Fig. S4B). In parallel, StUBC18 did not interact with either split-ARM motifs (ARM1, ARM2, ARM3, ARM4) or StPUB40_89–210_ (Fig. S4B). This suggests that both the U-box domain and ARM motifs confer on StPUB40 pairing specificity with StUBC18, similar to PUB22, while individual ARM motifs (ARM1, ARM2, ARM3, ARM4) have no binding activity (Fig. S4B and C).

### StPUB40 negatively regulates drought stress tolerance

**Figure 3:**
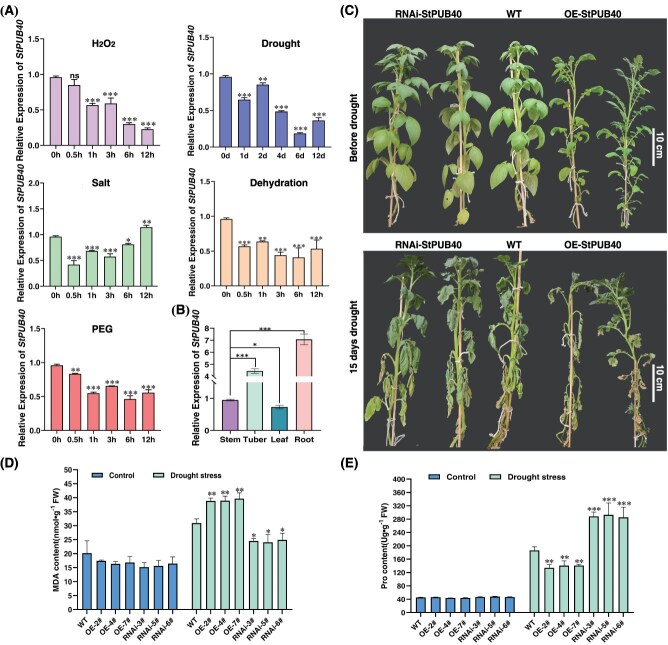
Characterization analysis of StPUB40. (A) The expression of *StPUB40* under different stress. (B) The organ-specific expression of the *StPUB40* in potato. (C) Phenotype of transgenic potato plants from restriction watering 15 days to 3 days rewatering. (D) The change of transgenic potato plants MDA content under drought stress. (E) The change of transgenic potato plants Pro content under drought stress. Asterisk marks above bars indicate significant differences (The data were determined by ANOVA followed by Tukey *post hoc* test, *n* = 3, ^*^P < 0.05, ^**^P < 0.01, ^***^P < 0.001).

To elucidate the functionality and mechanism of StPUB40 in potato, we detected *StPUB40* transcript levels during several treatments and tissue specificity by (qRT-PCR). The gene expression level of *StPUB40* exhibited differential gene expression patterns during various treatments. *StPUB40* was significantly downregulated under H_2_O_2_/drought/salt/PEG6000/dehydration stress (Fig. 3A). Moreover, the gene expression level of *StPUB40* was the highest in root, followed by tuber, stem, and leaf (Fig. 3B). Thus, *StPUB40* is involved in development and response to abiotic stress. In order to further investigate the biological functions of *StPUB40* responding to drought stresses in potatoes, the transgenic potato lines with overexpression (OE) and interference (RNAi) of *StPUB40* were established for further study (Fig. S5A and B). The results showed that OE plants were more sensitive to the effects of drought stress (Fig. 3C). Instead, RNAi plants exhibited increased drought stress tolerance (Fig. 3C). In addition, the MDA content in OE plants was markedly higher than that in RNAi plants under drought stress (Fig. 3D). Furthermore, Pro content in OE plants, meanwhile, was significantly lower than that in RNAi plants (Fig. 3E). Overall*, StPUB40* is a negative regulatory factor for potato drought tolerance.

### The complex of E2-E3, StUBC18-StPUB40, influences phenotype of potato plants

**Figure 4:**
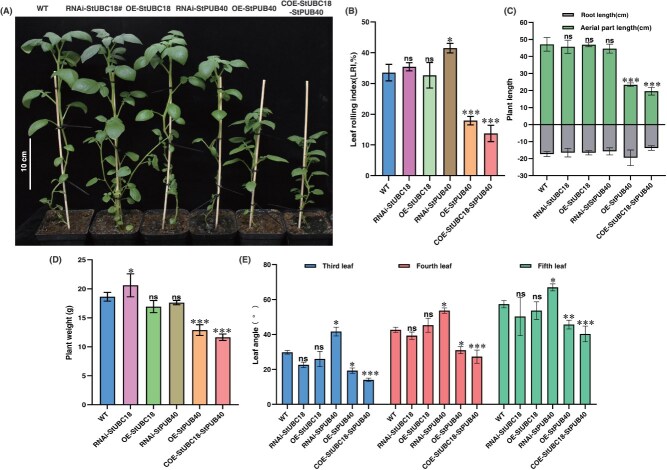
The function in the regulation of StUBC18-StPUB40 pair in the phenotype of potato plants. (A) Phenotypes of transgenic plants compared with WT plants. (B–E) Leaf rolling, plant length, plant weight, and leaf angle phenotypes of transgenic and WT plants. Asterisk marks above bars indicate significant differences (The data were determined by ANOVA followed by Tukey *post hoc* test, *n* = 3, ^*^P < 0.05, ^**^P < 0.01, ^***^P < 0.001).

To further evaluate the function of the StUBC18-StPUB40 pair in potato, the pair was co-overexpressed in potato cultivar ‘Atlantic’ (COE). The expression levels of *StUBC18* and *StPUB40* of all transgenic plants were detected by qRT-PCR (Fig. S6A). Phenotypic analysis of WT, OE-StPUB40, RNAi-StPUB40, and COE plants revealed pleiotropic phenotypic alterations. The COE plants displayed a more pronounced compact-type and erect stem phenotype than WT and OE plants (Fig. 4A). Furthermore, co-overexpression of *StUBC18* and *StPUB40* normally inhibited downward curling of the leaflets blade (called rolled leaf) and displayed a flat-leaf phenotype, while WT and OE plants exhibited slightly rolled leaves (Fig. 4A and B). In addition, COE plants had an enhanced erect-leaf habit and promoted an overall erect-leaf canopy. The plant weight and plant length also decreased in COE and OE plants compared to WT plants (Fig. 4C and D). Interestingly, co-overexpression of *StUBC18* and StPUB40 also affected the size of the leaf angle. We measured the leaf angle from the third leaf to the fifth leaf (from top to bottom). The result showed that leaf angle gradually increased (third leaf to the fifth leaf) in the COE plants (Fig. 4E). Nevertheless, the leaf angle of COE plants was substantially smaller than that of WT at the same position. Although we do not know whether the unrolled leaf resulted in compact-type plant phenotype, co-overexpression of *StUBC18* and *StPUB40* influences the plant type and leaf rolling phenotype.

### The StUBC18-StPUB40 pair is involved in drought stress tolerance

To further confirm whether the StUBC18-StPUB40 pair contributes to drought stress response, COE transgenic lines were chosen for further study. Notably, the agronomic traits of the COE plants were significantly different from OE-StPUB40 (OE) and WT plants under normal conditions.

**Figure 5:**
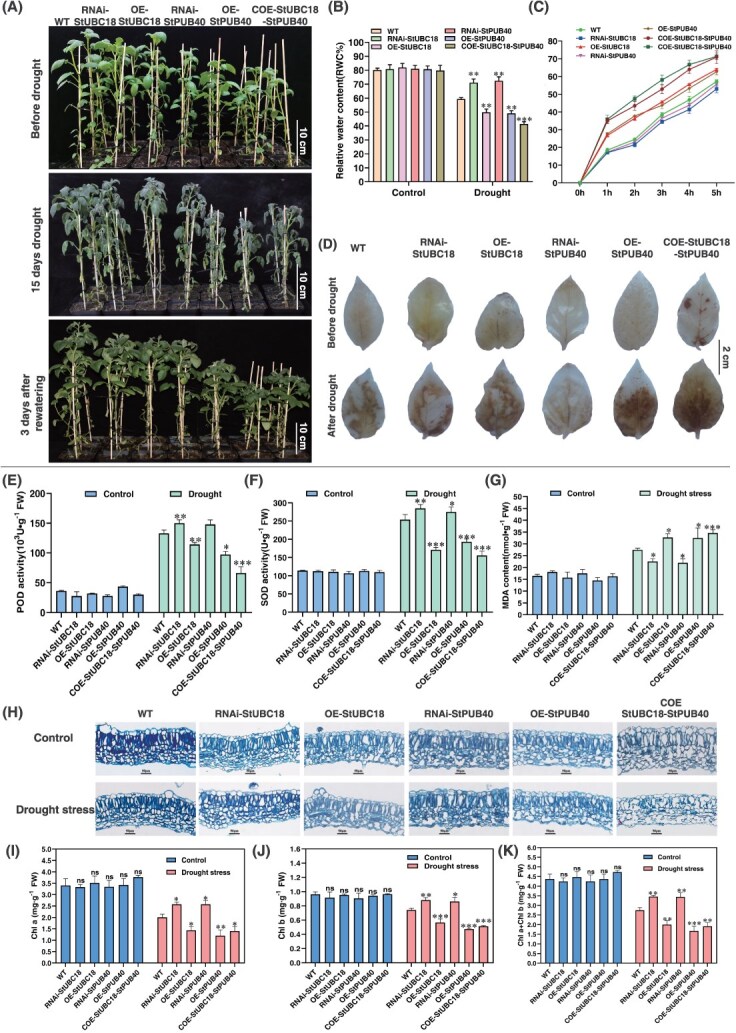
Phenotype of transgenic potato plants under restricted watering regimes. (A) Phenotype of OE-StUBC18, RNAi-StUBC18, OE-StPUB40, and RNAi-StPUB40 plants from restriction watering 15 days to 3 days rewatering. (B, C) The change of transgenic potato plants RWC and water loss rate under drought stress. (D) Detection of superoxide in OE-StUBC18, RNAi-StUBC18, OE-StPUB40, and RNAi-StPUB40 plants under control and water-stressed by DAB staining. (E–F) The SOD/POD activity and MDA content in potatoes after drought stress. (H) Altering of leaf anatomy in potato under drought stress. Scale bar = 50 μm. (I–K) Changes in chlorophyll content in potato under drought stress. Asterisk marks above bars indicate significant differences (The data were determined by ANOVA followed by Tukey *post hoc* test, *n* = 3, ^*^P < 0.05, ^**^P < 0.01, ^***^P < 0.001).

The expression levels of *StUBC18*/*StPUB40* of all transgenic plants were detected by qRT-PCR (Fig. S4A). Three significantly increased independent transgenic lines were chosen for further study. Mannitol stress severely affects plant rooting by reducing the number of lateral roots, root development by shortening the root length, and plant growth by limiting the increase in plant height in OE and COE plants compared to WT (Fig. S6B). Consistently, COE plants exhibited a weaker phenotype than WT plants under mannitol treatment. Mannitol treatment also caused the COE plants to wilt, with some leaves drying and showing a chlorosis phenotype. Consistent with the decrease in mannitol stress tolerance, COE plants had significantly more delayed development than OE, RNAi, and WT plants, especially a growth-inhibiting effect on the plant height (Fig. 5A). All plants, regardless of genotype, were observed to have a withering phenotype under drought stress (Fig. 5A). Moreover, COE plants dried up more severely than these WT, RNAi, and control plants when plants encountered 14-day drought stress. Our data suggested that co-overexpression of the StUBC18-StPUB40 pair led to significantly decreased drought tolerance in potatoes.

To further explore the physiological and biochemical mechanisms of drought tolerance affected by the StUBC18-StPUB40 pair, we measured all plant water loss, MDA, SOD, and POD activity. Water loss was drastically increased in all plants, while RWC (Relative Water Content) declined severely, especially in the COE plants (Fig. 5B and C). Diaminobenzidine (DAB) staining showed that co-overexpression of the StUBC18-StPUB40 pair in potato caused a greater accumulation of reactive oxygen species (ROS) than in those WT, RNAi, and OE plants under drought stress (Fig. 5D). In addition, superoxide dismutase (SOD) and peroxidase (POD) concentration significantly and rapidly decreased in COE plants, which indicated that co-overexpression of the StUBC18-StPUB40 pair affected scavenging capability of ROS-scavenging enzymes (Fig. 5E and F). However, the MDA content in COE plants was significantly higher than that in WT, OE plants, and RNAi plants (Fig. 5G). The data show that the StUBC18-StPUB40 pair negatively regulates drought stress response in potatoes.

To explore whether the StUBC18-StPUB40 pair also has an effect on the internal structure of the leaf at the cellular and tissue levels, an anatomical analysis was performed to observe the changes in the COE plant cross-section of a leaf. The palisade cells of WT and OE plants (OE-StPUB40) were arranged closely, and cell morphology was almost the same (Fig. 5H). In contrast, palisade cells of the COE plants were disorganized, irregular in appearance, loosely arranged, and of different sizes. Similarly, the spongy mesophyll of COE plants showed an irregular shape and unclear layers, and were arranged sparsely, with a larger intercellular space, while OE plants were consistent with that of WT. The ratio of palisade parenchyma to spongy parenchyma in WT and OE plants was greater than that of COE plants. In addition, the StUBC18-StPUB40 pair suppressed the palisade/spongy cells’ normal elongation resulting in changes. The palisade and spongy tissue thickness of WT are thicker than COE and OE plants (Fig. 5H, Table S3). The palisade and spongy tissue of COE plants are more irregular and disorganized under drought stress compared with normal conditions. The thickness of leaves significantly decreased, and the palisade and spongy tissue of COE and OE plants became thinner than that of WT under drought stress (Fig. 5H). Moreover, drought stress caused a significant decrease in the chlorophyll content of the COE plants compared with WT and OE plants (Fig. 4I–K).

### The StUBC18-StPUB40 pair has effects on belowground tuber yield

**Figure 6:**
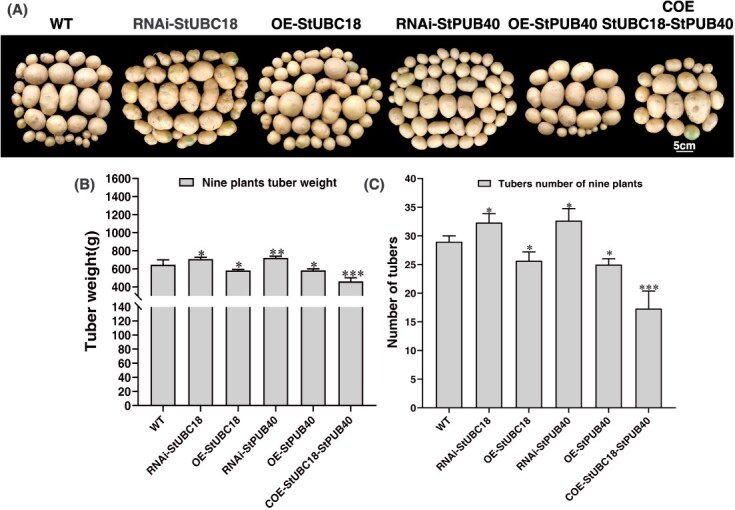
The StUBC18-StPUB40 pair negatively regulates potato tuber yield. (A) The morphology of tubers collected from transgenic potato plants. (B) The tuber weight of tuber in potato plants. (B) The numbers of tuber in potato plants. Asterisk marks above bars indicate significant differences (The data were determined by ANOVA followed by Tukey *post hoc* test, *n* = 9, ^*^P < 0.05, ^**^P < 0.01, ^***^P < 0.001).

Co-overexpression of StUBC18-StPUB40 pair in potato caused pleiotropic phenotypic alterations. It is plausible to assume that the StUBC18-StPUB40 pair has impacts on belowground tuber yield. To test this hypothesis, these plants (WT, OE, and COE) were grown in a greenhouse nursery, and tuber yield and number were assessed. The result showed that total tuber weight and tuber number per plant of RNAi plants were highest, followed by tuber yield with OE plants, with COE plants least (Fig. 6A–C). These results suggested that the StUBC18-StPUB40 pair was involved in the regulation of tuberization, influencing tuber number and tuber yield. Therefore, the StUBC18-StPUB40 pair is a negative regulator of tuber yield.

## Discussion

### StUBC18 interacts with StPUB40 to form StUBC18-StPUB40 pair regulating potato drought stress tolerance

Drought stress is the major factor that limits the development and growth of the potato. Here, we found that an E2 enzyme, StUBC18, is a negative regulator of drought tolerance. *StUBC18* exhibited a tissue-specific expression under normal conditions and was downregulated upon drought/salt stress (Fig. 1A and B). StUBC18 is categorized as class I because of its conserved domain UBC (Fig. 1C), which is similar to its closest homolog, AtUBC18 [26]. Phylogenetic analysis showed that the evolutionary tree of UBC18 was clustered into two branches. AtUBC18 belongs to a single branch (Fig. 1D). Sequence analysis indicated that UBC18 is highly conserved across multiple plants, which is likely to play crucial functions (Fig. 1D). As expected, overexpression of *StUBC18* increased the levels of MDA (Fig. 1H) and resulted in more severe wilting than WT plants (Fig. 1F). These results suggest that overexpression of *StUBC18* decreased drought tolerance in potatoes and Arabidopsis (Fig. 1F), similar to AtUBC18 in the situation in Arabidopsis [22]. StUBC18 is a negative regulatory factor for potato drought tolerance and several UBCs (including AtUBC1/AtUBC2, GmUBC9, AtUBC27, and AtUBC32) are involved in responding to drought tolerance [15, 17, 27, 28]. However, how StUBC18 contributes to drought stress tolerance remains unclear.

Considering that the E2-E3 pair plays a significant role in UPS, we screened 19 E3s interacting with StUBC18 (Table S2, Fig. 2A), including RGLG1/RGLG2/AIRP3/XBAT35 (associating with ABA signaling), MIEL1 (involved in auxin metabolism and ABA signaling), and PUB40 (relating with BR signaling). Those E3s have been reported to mediate the degradation of specific protein targets involved in abiotic stress resistance and signal transduction (Fig. 3B and Fig. S3A). For example, MIEL1 mediates MYB30 proteasomal degradation to weaken plant defense in Arabidopsis [29]. Evidence shows that U-box E3 ligase enzymes are implicated in many cellular processes, such as responses to biotic and abiotic stresses, regulating growth and development, and affecting signal transduction [30]. In this study, Y2H, BiFC, and SLC were performed to verify the interaction between StUBC18 and StPUB40 (Fig. 2B, C, F). StUBC18 interacts with the U-box and ARM domain of StPUB40, similar to reports for AtPUB13 [31].

Our previous study identified 66 U-box-type E3 ubiquitin ligases in potatoes and found 25 differential ubiquitination modification sites under drought stress [24]. It is noteworthy that StPUB27, the AtPUB19 homolog, negatively regulates drought stress tolerance by controlling stomatal conductance [25], suggesting that U-box ubiquitin ligases are implicated in drought stress. Surprisingly, co-overexpression plants of the StUBC18-StPUB40 pair increased the drought susceptibility of plants and displayed more serious wilted phenotypes than WT and OE plants (Fig. 5A). Drought stress triggers ROS production and excessive accumulation of ROS in plants, which is harmful to the cellular structure of the plants, and death can occur [32]. The ROS scavenging enzymes (SOD, POD) can convert excess ROS to reduce the risk of ROS injury [33]. MDA, a valid indicator of cytomembrane oxidative damage, indirectly reflects the extent of damaging membrane permeability [34]. In this study, the StUBC18-StPUB40 co-overexpression plants had lower SOD and POD activities than WT and OE plants (Fig. 3C), while the MDA contents were significantly higher than those of WT and OE plants (Fig. 4E–F). This indicates that co-overexpression of the StUBC18-StPUB40 induces excessive accumulation of ROS and is seriously harmful to membrane permeability and exacerbates plant senescence and death. Similarly, the group III E2 members in tomato and tobacco are associated with ROS production [35, 36]. Taken together, our data show that the StUBC18-StPUB40 pair negatively regulates drought stress tolerance in potato.

### The StUBC18-StPUB40 pair negatively regulates potato drought stress tolerance by altering leaf structure

Leaves are crucial organs in plants, primarily responsible for photosynthesis and exchanging gases. Leaf angle, a key agronomic trait in architecture, is mainly modulated by the BR signal [37]. The transcription factor BES1/BZR1, a master regulator, functions in phenotypes with erect leaves and semidwarfism [38]. In general, BZR1/BES1 is considered to regulate cell elongation and plant growth and respond to environmental stress [39, 40]. Several studies have reported that BZR1 is a target for the E3 ligase, modulating its stability by ubiquitin-mediated degradation, including StPUB40 [41–44], which regulated leaf angle. In this work, co-overexpression of StUBC18-StPUB40 pair decreased leaf angle at the same position compared to WT and OE plants (Fig. 4E), influenced the plant height (Fig. 4C), and produced a compact and dense plant type. In addition, the StUBC18-StPUB40 pair also influences plant weight (Fig. 4D).

Leaf rolling, a frequently observed phenotype in cereals, is an important agronomic trait for high yield [45]. The appropriate leaf rolling improves photosynthetic efficiency and regulates water loss by decreasing leaf transpiration under drought stress [46]. Research shows that leaf rolling is usually regulated by altering bulliform cell number or shrinking and expanding [45]. At the molecular level, the antagonistic effects between small RNAs and their target transcription factors and genes controlling leaf rolling (ROLLED LEAF genes) are key factors regulating leaf rolling [47]. Some research has suggested that the E3 enzyme is related to modulating leaf rolling, such as OsRINGzf1 [48] and Drought hypersensitive (DHS) [49]. However, the molecular and genetic mechanisms of leaf rolling remain to be elucidated in potatoes. In this study, plants co-overexpressing *StUBC18* and *StPUB40* were more dramatically rolled, exhibiting flat leaves compared with WT and OE plants under normal conditions, suggesting that the StUBC18-StPUB40 pair inhibit appropriate leaf rolling (Fig. 3A and B). Although we do not know whether the changes of palisade cells directly influence leaf growth and development, the StUBC18-StPUB40 pair alters the ratio of palisade parenchyma to spongy parenchyma, which may be regulating key drought stress tolerance elements.

Leaf rolling helps to promote the formation of thicker palisade tissue and epidermis [50]. Histological analysis showed that the StUBC18-StPUB40 pair influences palisade cell arrangement and size (Fig. 3J). The change in leaf structure caused the change in physiological function under drought stress [51]. The highly developed palisade tissue plays a dual role in protecting mesophyll cells from being damaged by strong light under drought stress and exploiting diffracted light for photosynthesis. This characteristic facilitates higher levels of photosynthetic capacity, allowing for deeper light penetration and improved CO_2_ diffusion, thus allowing for better photosynthesis [52, 53]. The development of the mesophyll palisade tissue, an increase in the number of cell layers, and a decrease in the volume, as well as a relative reduction in the amount of spongy tissue, are all responses of plants to water deprivation [54]. The greater the density of arrangement, the better the plants utilize light energy [50]. In this study, our data showed that the palisade and spongy tissue thickness are significantly decreased under drought stress, with the greatest reduction in COE plants and the least in WT plants (Fig. 5H, Table S3). Moreover, changes in sponge and palisade tissue had a significant impact on chlorophyll content in leaves under drought stress, which is one of the major factors affecting photosynthesis and drought resistance of plants.

### The StUBC18-StPUB40 pair negatively regulates tuber yield

The potato tuber, a specialized stem, is considered to arise from the stolon, a subterranean organ [55]. Previous reports suggested that tuber formation and development are influenced by factors such as photoperiod, temperatures, and drought stress. There are three key regulation factors for major signals inducing tuber formation: StCDF1, StSP6A (SELF-PRUNING6A), and StBEL5 [56]. Recent studies have revealed that StPHYF has a critical role in potato photoperiodic tuberization by stabilizing the StCOL1 protein, which regulates the CO-FT tuberization pathway [57]. In this work, we verified that co-expression of the StUBC18-StPUB40 pair led to decreased tuber yield and influenced tuber number (Fig. 6A–C). Numerous investigations in other plants have indicated that BZR1, a target protein of PUB40, is related to grain size and the number of seeds [58].

### A model for how the StUBC18-StPUB40-target proteins complex exerts its functions

**Figure 7:**
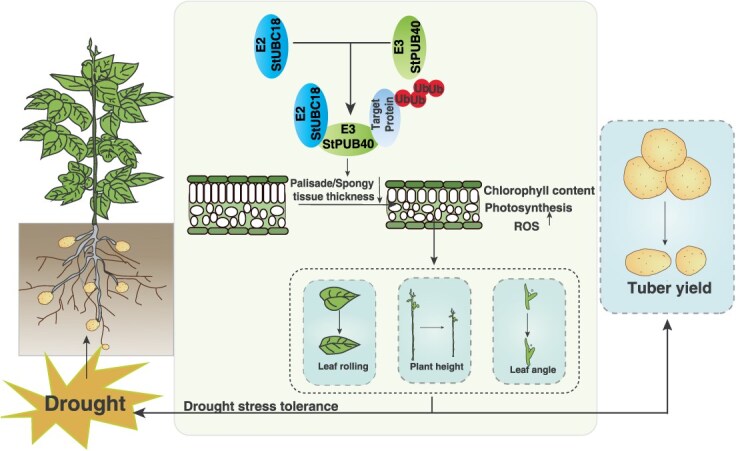
Putative working model of StUBC18-StPUB40 pair. The StUBC18-StPUB40 pair is involved in regulating drought stress by altering mesophyll thickness.

Given that E2 and E3 regulate the diverse cellular processes, describing the function for cooperation function of a chimeric E2-E3 enzyme is essential for studying the UPS mechanism degradation of the target protein. Based on previous research and the present study, we proposed a working model of StUBC18-StPUB40-target proteins: StPUB40 interacts with StUBC18 and subsequently conjugates with target to form a complex StUBC18-StPUB40, which mediates target proteins ubiquitination and degradation (Fig. 7). The low-abundance target protein influences plant development and drought stress tolerance. Support for such a model is presented by the fact that the StUBC18-StPUB40 pair influences the phenotype of potato plants and negatively regulates drought stress tolerance.

Despite significant advances in understanding the role of the StUBC18-StPUB40 pair, there are still several important concerns. How does the potato employ the StUBC18-StPUB40 pair’s multiple functions? StUBC18 can interact with several E3 ligases that regulate various signals (Fig. S3D). How do the multiple StUBC18-E3 pairs achieve exact and accurate regulation of crosstalk between many signals and balance multiple signal transduction? These intriguing questions should be explored in further studies.

## Conclusion

Our study offers a novel insight into a mechanism of StUBC18-StPUB40 pair that regulates drought stress tolerance, development, and tuber yield by influencing pleiotropic alterations. This research will provide a crucial theoretical foundation for oriented genetic improvement of novel potato varieties that are drought-resistant, high-yielding, and high quality.

## Materials and methods

### Plant growth conditions and treatments

Nontransgenic potato plants (*Solanum tuberosum* cultivar Atlantic) and transgenic plants were cultured on Murashige and Skoog (MS) media with 3% sucrose solidified with 0.75% agar, and grew for 4 weeks under 16 h light/8 h dark photoperiod at 22°C with 50% relative humidity. Four-week-old plants were sown in plastic pots (10 × 10 cm) supplemented with a steam-sterilized soil mix (3:1, soil:vermiculite) and transferred to a growth chamber under 16 h light/8 h dark regime and watered weekly. After 1 month of growth, the plants were divided into three treatment groups. For salinity treatment, the plants were irrigated with 200 mM NaCl or water (control), and the samples were harvested at 0, 0.5, 1, 3, 6, and 12 h; for the drought stress treatment, the plants were withheld irrigation for ~2 weeks and sampled at a field capacity of 35%–45%, and then rewatered for 3 days, while controls were watered weekly and samples were collected at the same point.

### Sequence alignment and phylogenetic analysis

The StUBC18 homologs sequence from *Arabidopsis thaliana*, *Populus trichocarpa*, *Solanum lycopersicum*, *Oryza sativa*, *Hordeum vulgare*, *Zea mays*, *Vitis riparia*, and *Medicago truncatula* were searched and obtained from Phytozome database (https://jgi.doe.gov/), and aligned using ClustalW with the default parameters. The Phylogenetic analysis was estimated with MEGA X software using the neighbor-joining method, and the bootstrap values were set to 1000 replicates. Amino acid sequences and conserved structures were analyzed by DNAMAN.

### RNA isolation and qRT-PCR

Total RNA from the above-mentioned samples was extracted using RNAprep Pure Plant Kit (Tiangen Biotechnology, China) and cDNA was synthesized using Quantscript Reverse Transcriptase Kit (Tiangen Biotech) according to the manufacturer’s instructions. Quantitative real-time PCR (qRT-PCR) was carried out using Fast Fire qPCR PreMix (Tiangen Biotechnology, China) with LightCycler® 96 System. The potato *ef1α* gene (GenBank accession: AB061263) served as the internal control [59]. The transcript abundance was calculated using the 2^-△△CT^ method [60]. All specific primers used for qRT-PCR are shown in Table S1.

### Gene cloning, plasmid construction, generation of transgenic plants, and treatments

The entire coding region of *StUBC18* and *StPUB40* was cloned from Atlantic cDNA using specific primers listed in Table S1. The binary vector pRI 201-AN (TaKaRa Biotechnology, Dalian, China) vector was used to construct an overexpression vector, which contains two MCSs. The *StUBC18* and *StPUB40* were inserted into pRI201-AN DNA vector according to the manufacturer’s instructions and pRI201-35S:*StUBC18*, pRI201-35S:StPUB40, pRI201-35S:*StUBC18*-35S:*StPUB40*, pBI121-StUBC18-RNAi, and pBI121-StPUB40-RNAi were obtained. The constructed vectors were transferred to *Agrobacterium* strain GV3101. Potato cultivar ‘Atlantic’ plants were used to generate transgenic plants in this study. The *Agrobacterium*-mediated transformation of microtubers was used to generate transgenic potato plants, which was performed as previously described by Si *et al* [61]. The plants were screened on MS with 50 μg/ml kanamycin resistance and determined transformed plants by PCR detection. For potato phenotypic observation, the transgenic plants grew under the conditions described above. After 2 months, leaf rolling and leaf angle were measured and counted. For drought stress, the transgenic potato plants treatment was performed as described above.

### Subcellular localization and colocalization

The full-length coding sequences (CDS) of *StUBC18* and without the stop codon were cloned from potato and recombined into pCAMBIA1300-EGFP vector for the generation of EGFP fused target protein (StUBC18), respectively. The *StPUB40* CDS without the stop codon was cloned into a pCAMBIA1300-mCherrry vector. The recombinant plasmids were transferred into *Agrobacterium* strain GV3101. For Subcellular localization, the *Agrobacterium tumefaciens*-harboring fusion constructs were injected into *N. benthamiana* leaves. For colocalization, the different indicated combinations were transiently expressed in *N. benthamiana* leaves. After 48–72 h, the GFP and mCherry fluorescence were visualized using an inverted confocal microscope (Zeiss LSM780, Germany), respectively. Primers used for localization were mentioned in Table S1.

### Yeast two-hybrid assays and screening

The Y2H assays were carried out as described previously [62]. The CDS of *StUBC18* was cloned and fused to pGBKT7 as bait. The cDNA library as prey and the bait pGBKT7-StUBC18 were cotransformed into yeast strain YH109 using the PEG/LiAc method. The interaction proteins screening was performed according to the supplier’s instructions (Clontech) via growing on SD/−T–L, SD/−T–L–H, and SD/−T–L–H–A selective dropout medium to confirm protein interactions. One-to-one Y2H was used to confirm protein interactions and detected by the β-galactosidase quantitative assays [63]. The StPUB40 interaction protein was screened using the dual split-ubiquitin membrane Y2H system [64]. The detailed procedure of StPUB40 interaction protein screening was mentioned above.

### Bimolecular fluorescence complementation analysis and split-luciferase complementation assay

BIFC analyses were performed as described previously [65]. The coding sequence of *StUBC18* and *StPUB40* was separately integrated into pSPYCE-35S and pSPYNE-35S vectors and further transferred into *Agrobacterium* strain GV3101. The YFP fluorescence signal was observed and imaged using an inverted confocal microscope (Zeiss LSM780, Germany) 48–72 h after infiltration. SLC assays were carried out as described previously [66]. StUBC18and StPUB40 coding sequences were separately cloned into pCAMBIA1300-CLuc and pCAMBIA1300-NLUC, respectively, and transferred into *Agrobacterium* strain GV3101. The different combinations were co-injected into *N. benthamiana* leaves. The LUC signal was observed and captured using PlantView 100 (Guangzhou Biolight Biotechnology, China).

### DAB staining

Histochemical staining (DAB staining) was used to determine superoxide (H_2_O_2_), the detailed experimental procedure described as previously [51]. Detached leaves were immersed and infiltrated with 1 mg/ml DAB solution at 37°C in the dark for 16 h. The samples were boiled using a bleaching solution (ethanol:acetic acid = 3:1) at 95°C for 15–20 min after the end of staining and followed by decolorizing with 95% ethanol to bleach out the chlorophyll (decolorization step). The reaction between DAB and H_2_O_2_ caused brown precipitate on leaves.

### Measurement of RWC, MDA, SOD, and POD

The RWC assay was measured according to the method described previously by Verslues *et al* [67]. Briefly, the first fully expanded leaf was weighed to obtain its fresh weight (FW). Then, it was hydrated with distilled water for 12 h to determine the turgid weight (TW). After that, it was dried at 65°C until a constant weight was reached to measure the dry weight (DW). The RWC was calculated as follows: RWC (%) = (FW–DW)/(TW–DW) × 100%.

The measurement of MDA content was performed according to the method described by Heath [68]. SOD activity measurement is based on the method of Beyer and Fridovich [69]. The measurement of POD activity referred to a method of Wang [70]. The three technical replicates were conducted for each of the three biological replicates.

## Supplementary Material

Web_Material_uhaf145

## Data Availability

All relevant data can be found within the paper and its supporting materials.
