# Supplementary material for: The StUBC18-StPUB40 pair negatively regulate drought stress tolerance and influences tuber yield in potato
Source: Hortic Res. 2025 Jun 10;12(9):uhaf145. doi: 10.1093/hr/uhaf145 (PMC12313339; doi:10.1093/hr/uhaf145)
Supplement: Web_Material_uhaf145 [file web_material_uhaf145.zip › Supplementary data-Figure.docx]

**
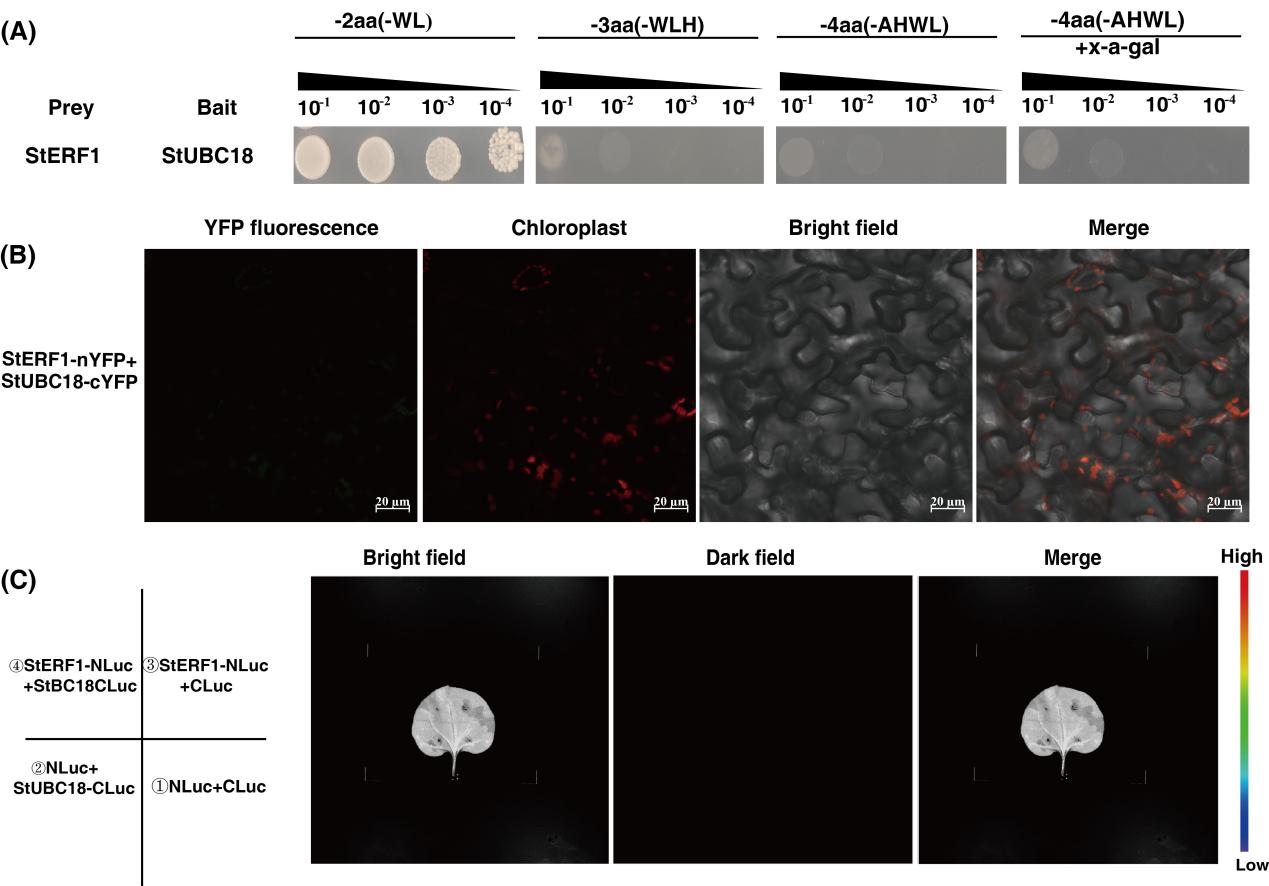
**

**Figure S1 The interaction beteween StUBC18 and StERF1.** (A) Yeast two-hybrid assays of the interaction between StUBC18 and StEFR1. (B) Verification of the interaction between StUBC18 and StEFR1 by BiFC assay. (C) Verification of the interaction between StUBC18 and StEFR1 by SLC assay.

**
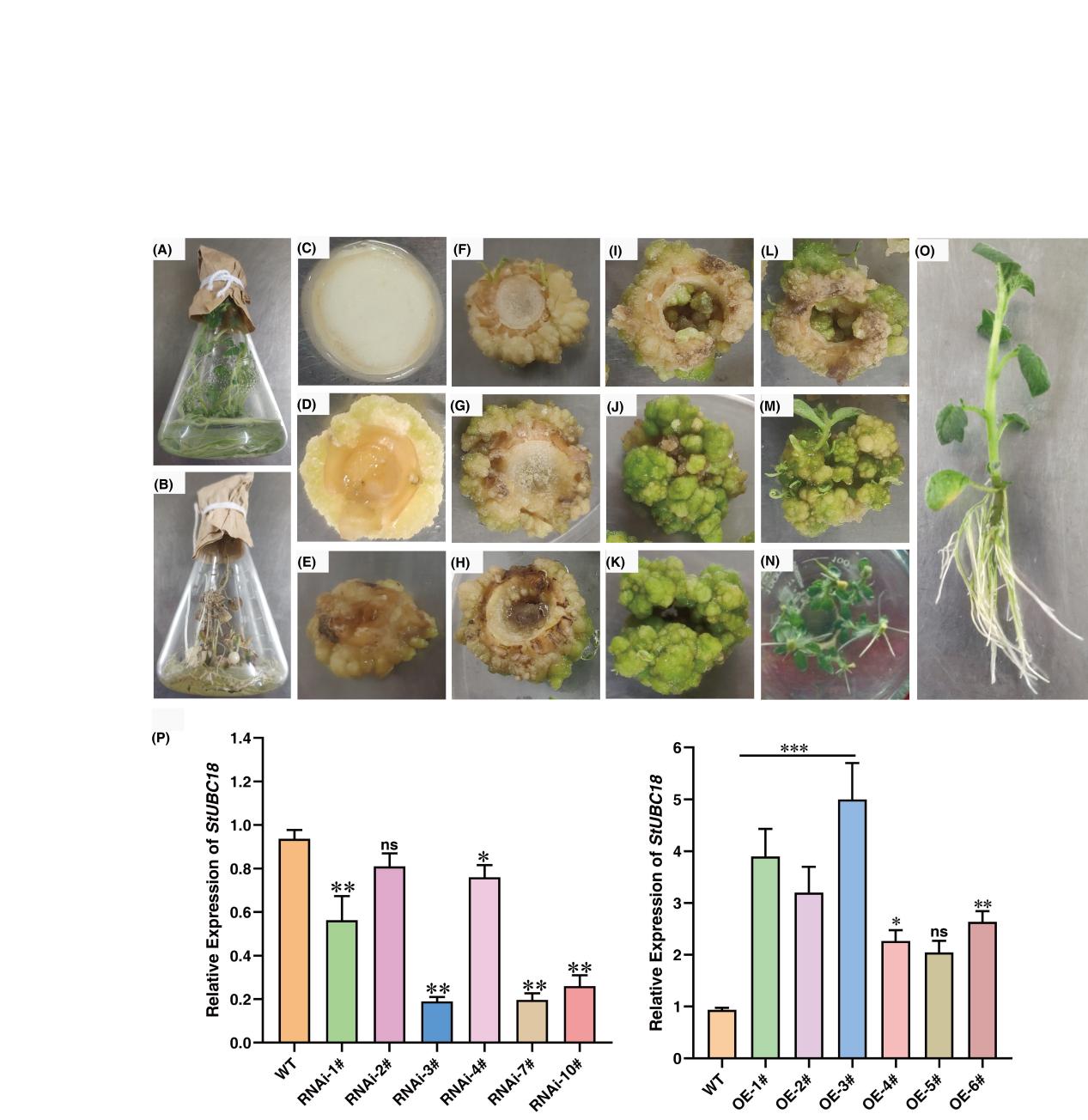
**

**Figure S2 The transgenic plant regenerated from microtuber by agrobacterium transformation system.** (A-B) The potato microtuber inducement on MS medium with 8% sucrose. (C-M) The callus induction and redifferentiation of potato. (N-O) The regenerated fertile transgenic plants were obtained by effective selection for resistant screening. (P) The relative expression of *StUBC18 in* OE and RNAi were detected by qRT-PCR.

**
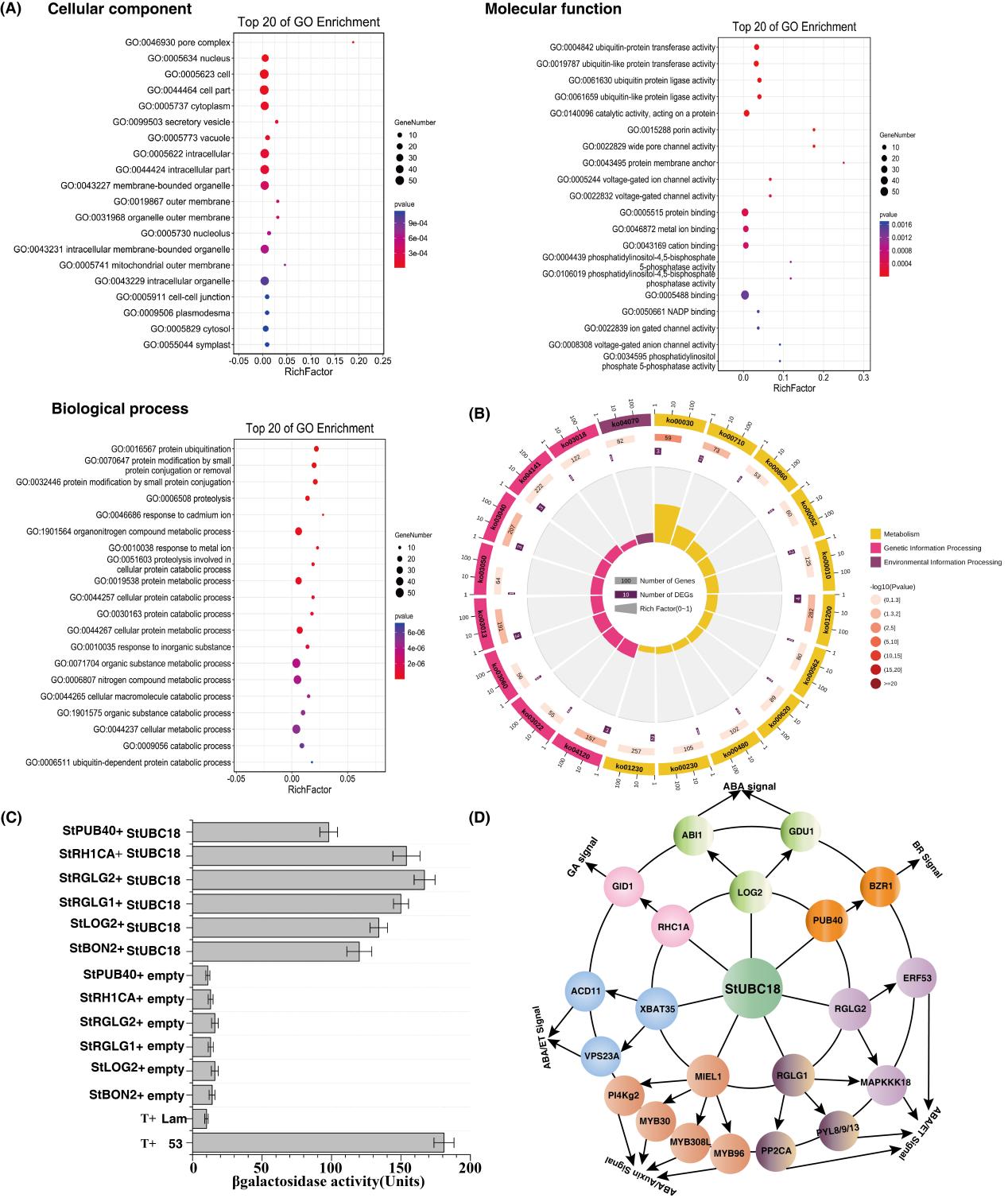
**

**Figure S3 The analysis of StUBC18 interaction protein.** (A) GO analysis including cellular component, molecular function, biological process. (B) KEGG analysis. (C) The StUBC18 interaction protein for β-galactosidase quantitative assays. (D) The substrate protein interaction network of StUBC18 interaction E3s.


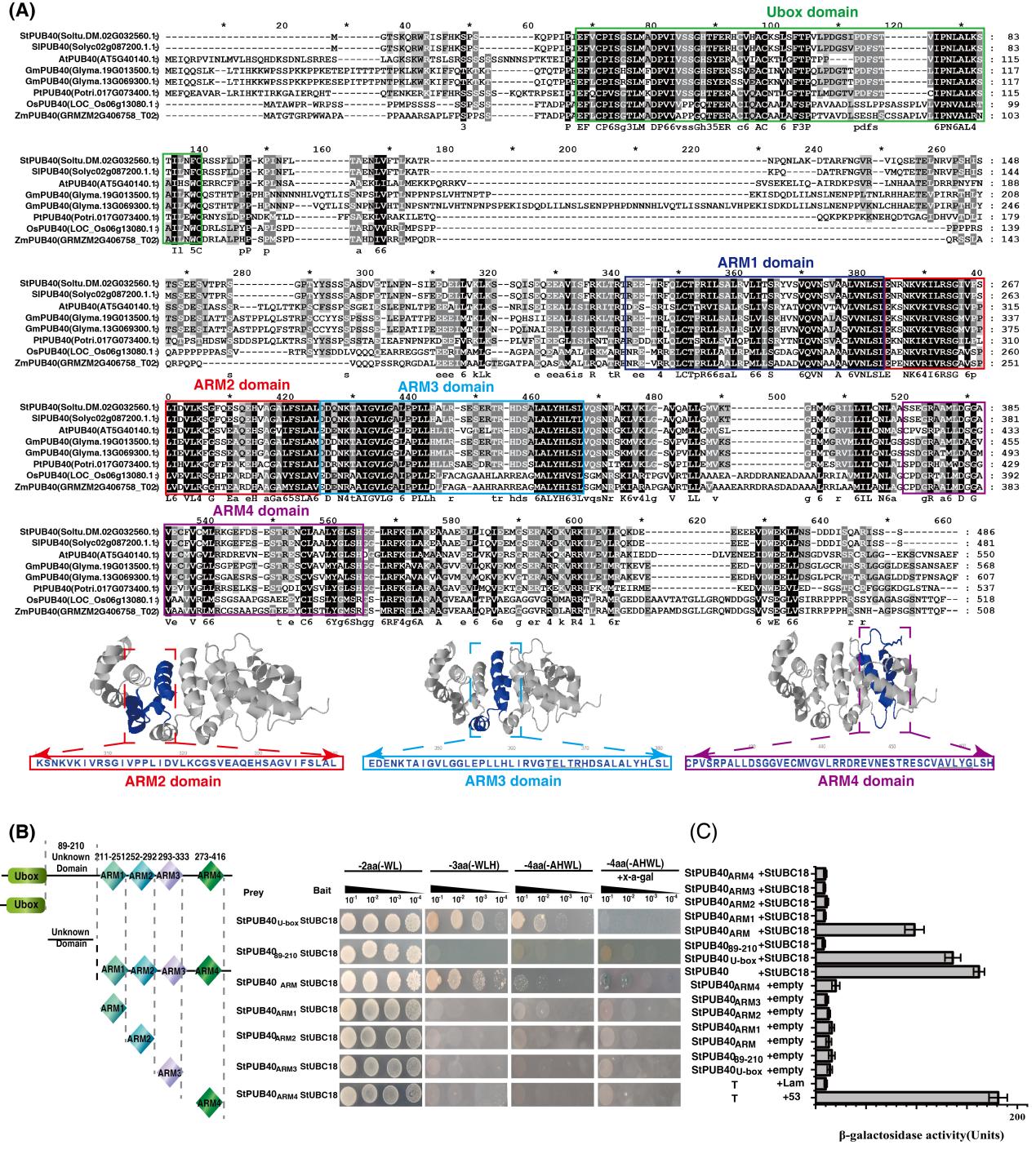


**Figure S4 Characterization and sequence analysis of StPUB40.** (A) Multiple alignments of an amino acid sequence of PUB40. (B) StUBC18 interacts with the C-terminal and N-terminal regions of StPUB40, including the U-box and ARM domain. Verification of U-box and ARM domain of StPUB40 interacts with StUBC18 by Y2H analysis. (C) The StPUB40 interaction domain for β-galactosidase quantitative assays.

**
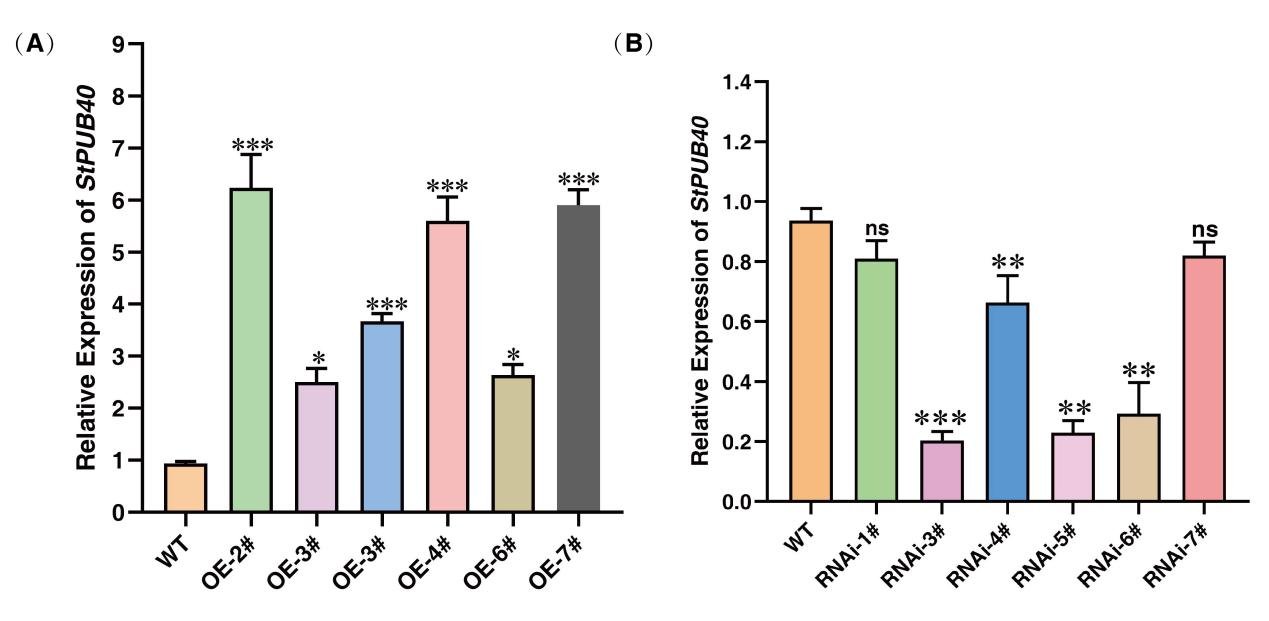
**

**Figure S5 The relative expression of *StPUB40* in OE and RNAi plants were detected by qRT-PCR.**

**

**

**Figure S6 Phenotypic and stress tolerance studies of COE plants.** (A) The expression of StUBC18 and StPUB40 gene in potato plants. (B) The phenotype of transgenic potato plants on MS supplemented with mannitol.
