# Supplementary material for: The StUBC18-StPUB40 pair negatively regulate drought stress tolerance and influences tuber yield in potato
Source: Hortic Res. 2025 Jun 10;12(9):uhaf145. doi: 10.1093/hr/uhaf145 (PMC12313339; doi:10.1093/hr/uhaf145)
Supplement: Web_Material_uhaf145 [file web_material_uhaf145.zip › Supplementary data-table.docx]

**Table S1 Sequences of the primers used in this study**

| **Gene** | **Forward primer sequence** | **Reverse primer sequence** |
| --- | --- | --- |
| Primers for real-time quantitative PCR | | |
| RT-UBC18 | TGGTTCCATGATGATAAGGTGT | TATTGGACAGTAAAGGCCAACT |
| RT-PUB20 | TGGACCCACTTATTACTCTTCG | GAAACGAGATGACAGCTTCTTC |
| RT-ef1α | ATTGGAAACGGATATGCTCCA | TCCTTACCTGAACGCCTGTCA |
| Primers for over-expression vector | | |
| PRI201-PUB40 | CACTGTTGATACATATGATGGGTACTAGCAAGCAAAGAT | ATTCAGAATTGTCGACTATGAAGAACTTATCCGTGCTTG |
| PRI201-UBC18 | CACTGTTGATACATATGATGTCCGCCTCCTCCGCCTC | ATTCAGAATTGTCGACTCACACCTTATCATCATGGAACCACCA |
| Universal primers for PRI201-AN | AN-M3:GAATTGGTACCTGCGGCCGCGTAAAACGACGGCCAGT | AN-RV:CAGGAAACAGCTATGACCATGATTACGAATTCCCGGG |
| Primers for yeast two-hybrid assays | | |
| GBKT7-UBC18 | AGGAGGACCTGGAATTCGGAAGCGGGGCCAATGAATA | CTGCAGGTCGACGGATCCGGCCAACTTGCTGCACATTA |
| pGADT7-BON1 | gccatggaggccagtgaattcATGCAGTTGTCTCTATCTGCTACCA | cagctcgagctcgatggatccTCACAAAGTCGGCTGTATCTTTCT |
| pGADT7-LOG2 | gccatggaggccagtgaattcATGGGTAATATGGGAAGTAGTGGTG | cagctcgagctcgatggatccTTACTCTTCGGCTGCGCCT |
| pGADT7-RHC1A | gccatggaggccagtgaattcATGTCGAGTGGGAGCTATACACAC | cagctcgagctcgatggatccTTAAAAGCGCCACGCAGG |
| pGADT7-RGLG1 | gccatggaggccagtgaattcATGGGGAATCAAGAGTCTGCC | cagctcgagctcgatggatccCTAGCTATATAGTCTTATGCGAGTTTTAATG |
| pGADT7-RGLG2 | gccatggaggccagtgaattcATGGGTGGCAAGAGTTCAAAGA | cagctcgagctcgatggatccTCAGTAAAGTCTTATCCTAGTTTGAATACTG |
| pGADT7-PUB40 | | |
| pGADT7-PUB40-U-box | GACGTACCAGATTACGCTCATATGGGTACTAGCAAGC | AGCTCGAGCTCGATGGATCCCTAACGGGTTCGGGTCAA |
| pGADT7-PUB40-_ARM_ | CCATGGAGGCCAGTGAATTCATGGAAGAAACCCGGTTC | CGATTCATCTGCAGCTCGAGCTATGAAGAACTTATC |
| pGADT7-PUB40_ARM1_ | ACGTACCAGATTACGCTCATATGCGTGAAGAAACCCGG | ATGCCCACCCGGGTGGAATTCCTATAACGACAAATTTAC |
| pGADT7-PUB40_ARM2_ | ACGTACCAGATTACGCTCATATGGAGAATCGAAACAAG | ATGCCCACCCGGGTGGAATTCCTATAACGCTAAACTGAAAAG |
| pGADT7-PUB40_ARM3_ | ACGTACCAGATTACGCTCATATGGATGATCAGAACAAAAC | ATGCCCACCCGGGTGGAATTCCTATAAAGAAAGATGATATAG |
| pGADT7-PUB40_ARM4_ | ACGTACCAGATTACGCTCATATGAGTTCTGAAGGGAGA | ATGCCCACCCGGGTGGAATTCCTAATGACTAAGTCCATAC |
| Primers for yeast three-hybrid assays | | |
| pBridge-UBC18 | TTGACTGTATCGCCGGAATTCATGTCCGCCTCCTCCG | TGGCTGCAGGTCGACGGATCCTCACACCTTATCATC |
| pBridge-UBC18-PUB40 | GAAGAGAAAGGTGGCGGCCGCAATGGGTACTAGCAAGC | AGATCTTCGGGCTAATGCGGCCGCCTATGAAGAACTTATCCG |
| Primers for subcellular localization | | |
| PUB40-mCherry | AGAACACGGGGGACTCTAGAATGGGTACTAGCAAGC | AGGGACTGACCACCCGGGGATGAAGAACTTATCCGTG |
| 18-EGFP | ACGGGGGACGAGCTCGGTACCATGTCCGCCTCCTCCG | CTCACCATGTCGACTCTAGACACCTTATCATCATG |
| Primers for BiFC | | |
| pSPYNE-PUB40 | GGCGCGCCACTAGTGGATCCATGGGTACTAGCAAGC | GCGGTACCCTCGAGGTCGACTGAAGAACTTATCCG |
| pSPYCE-UBC18 | GGCGCGCCACTAGTGGATCCATGTCCGCCTCCTCCG | GCGGTACCCTCGAGGTCGACCACCTTATCATCATG |
| Primers for SLC |  |  |
| PUB40-NLuc | cgagctcggtacccgggatccATGGGTACTAGCAAGC | cgcgtacgagatctggtcgacTGAAGAACTTATCCGTG |
| UBC18-CLuc | tacgcgtcccggggcGGTACCATGTCCGCCTCCTCCG | TGGCGCGCCGGGCCCTCTAGATCACACCTTATCATCATG |

**Table S2 Positive clones from yeast two-hybrid screening and information of StUBC18 interaction proteins**

| **Clone No.** | **Description** | **Potato code** | **AGI code** |
| --- | --- | --- | --- |
| 1 | Calcium-dependent lipid-binding (CaLB domain) family protein | Soltu.DM.06G013260.1 | AT5G23950 |
| 2 | Serine/Arginine-rich protein (SRP34A) | Soltu.DM.03G021660.2 | AT3G49430 |
| 3 | A voltage-dependent anion channel (VDAC2) | Soltu.DM.02G027730 | AT5G67500 |
| 4 | Alpha subunit of the elongation factor-1 complex (EF1ALPHA) | Soltu.DM.06G005680 | AT5G60390 |
| 5 | Outer envelope pore 24B-like protein | Soltu.DM.02G025400.1 | AT1G45170 |
| 6 | Chloroplast localized glyceraldehyde-3-phosphate dehydrogenase (GAPB) | Soltu.DM.04G037850.1 | AT1G42970 |
| 7 | RING/U-box superfamily protein | Soltu.DM.12G005790 | AT5G42940 |
| 8 | Calcium-dependent lipid-binding (CaLB domain) family protein (CBL1) | Soltu.DM.08G023660.2 | AT4G17615 |
| 9 | A subtilisin-like serine protease (ARA12) | Soltu.DM.02G027920 | AT5G67360 |
| 10 | Transcription initiation factor IIF | Soltu.DM.07G009560.1 | AT1G75510 |
| 11 | Member of Histidine Kinase, phytochrome E | Soltu.DM.02G014300.1 | AT4G18130 |
| 12 | Alba DNA/RNA-binding protein(ALBA1) | Soltu.DM.06G019430.1 | AT1G29250 |
| 13 | Heat shock protein (HSP22) | Soltu.DM.03G027830.1 | AT4G10250 |
| 14 | An ATP-dependent RNA helicase | Soltu.DM.06G006910.1 | AT5G11170 |
| 15 | Ubiquitin E3 ligase LOG2 (LOSS OF GDU2) | Soltu.DM.09G005970.1 | AT3G09770 |
| 16 | An RNA–binding protein involved in stress granule formation (UBP1B) | Soltu.DM.06G022260.2 | AT1G17370 |
| 17 | Eukaryotic translation initiation factor 4A-1(TIF4A1) | Soltu.DM.08G012960.1 | AT3G13920 |
| 18 | Idolase-type TIM barrel family protein | Soltu.DM.05G006110.3 | AT3G01850 |
| 19 | Cysteine proteinase precursor-like protein/ dehydration stress-responsive gene (RD21) | Soltu.DM.12G005890.1 | AT1G47128 |
| 20 | RING/U-box superfamily protein | Soltu.DM.03G028780.1 | AT5G08139 |
| 21 | E3 ubiquitin ligase for the GA-receptor GID1(RHC1A) | Soltu.DM.01G005890.1 | AT2G40830 |
| 22 | 26S proteasome AAA-ATPase subunit RPT3 (RPT3) | Soltu.DM.06G002730.1 | AT5G58290 |
| 23 | A cytosolic glucose-6-phosphate dehydrogenase(G6PD6) | Soltu.DM.02G033900.5 | AT5G40760 |
| 24 | ER membrane protein complex subunit-like protein | Soltu.DM.04G014700.1 | AT1G65270 |
| 25 | An RNA–binding protein involved in stress granule formation (UBP1B) | Soltu.DM.06G022260.2 | AT1G17370 |
| 26 | Phosphoinositide-specific phospholipase C (PLC2) | Soltu.DM.05G022790.1 | AT3G08510 |
| 27 | A protein with similarity to serine protease, subtilisin (SASP) | Soltu.DM.06G022310.1 | AT3G14067 |
| 28 | Vacuolar sorting receptors family (RMR2) | Soltu.DM.11G011840.1 | AT1G71980 |
| 29 | A mitochondrially targeted DNAJ protein (GFA2) | Soltu.DM.01G027970.1 | AT5G48030.1 |
| 30 | Phosphatase-related (SGT1B) | Soltu.DM.03G023300.1 | AT4G11260 |
| 31 | Ubiquitin ligase (XBAT35) | Soltu.DM.09G026770.2 | AT3G23280 |
| 32 | RING/U-box superfamily protein that impacts on auxin signaling output (GASP1) | Soltu.DM.01G039510.1 | AT3G05545 |
| 33 | CCCH-type/C3HC4-type RING finger | Soltu.DM.01G027650.1 | AT3G08505 |
| 34 | Hypothetical protein | Soltu.DM.07G004600.2 | AT1G16840 |
| 35 | A cytoplasmic MAP1 like methionine aminopeptidase (MAP1A) | Soltu.DM.01G031710.1 | AT2G45240 |
| 36 | Plant-specific putative DNA-binding proteins (PPD2) | Soltu.DM.06G034530.1 | AT4G14720 |
| 37 | A member of the Sad1/UNC-84 (SUN)-domain proteins (SUN1) | Soltu.DM.01G035950.1 | AT5G04990.1 |
| 38 | A phosphatidyl glycerophosphate (PGP) phosphatase (PTPMT1) | Soltu.DM.12G025590.1 | AT2G35680 |
| 39 | Arabidopsis homologs of the yeast/human Hrd1 protein (HRD1A) | Soltu.DM.03G021000.2 | AT3G16090 |
| 40 | A cytosolic phosphoglucomutase (PGM3) | Soltu.DM.04G015010.1 | AT1G23190 |
| 41 | A protein similar to JAB1(AJH1/CSN5A) | Soltu.DM.06G028470.1 | AT1G22920 |
| 42 | DCD (Development and Cell Death) domain protein | Soltu.DM.03G032460.2 | AT5G61910 |
| 43 | Polyubiquitin 10(UBQ10) | Soltu.DM.11G008460.1 | AT4G05320 |
| 44 | C2H2-like zinc finger protein | Soltu.DM.06G013220.1 | AT4G25610 |
| 45 | RING-type E3 ubiquitin ligase that interacts with and ubiquitinates MYB30(MIEL1) | Soltu.DM.06G015770.1 | AT5G18650 |
| 46 | CCCH-type/C3HC4-type RING finger | Soltu.DM.01G027650.1 | AT3G08505 |
| 47 | C3HC4 type (RING finger) family protein | Soltu.DM.02G001760.2 | AT5G23110 |
| 48 | Kunitz family trypsin and protease inhibitor protein | Soltu.DM.03G023510.1 | AT1G73325 |
| 49 | C3HC4-type RING finger E3 ubiquitin ligase | Soltu.DM.01G005600.1 | AT5G19430 |
| 50 | A RING-H2 protein that interacts with the RING finger domain of COP1(CIP8) | Soltu.DM.09G022870.1 | AT5G64920 |
| 51 | NBR1, a selective autophagy substrate (NBR1). | Soltu.DM.03G025860.1 | AT4G24690 |
| 52 | A small glycine-rich RNA binding protein (GRP7) | Soltu.DM.01G049440.1 | AT2G21660 |
| 53 | Luminal binding protein (BiP2) | Soltu.DM.03G022200.1 | AT5G42020 |
| 54 | Putative RNAse III-Like protein (NFD2) | Soltu.DM.08G019330.1 | AT1G12560 |
| 55 | RING domain ubiquitin E3 ligase, RGLG1(RING domain ligase 1) | Soltu.DM.09G020590.2 | AT5G63970 |
| 56 | RING domain ubiquitin E3 ligase, RGLG2 (RING domain ligase 2 | Soltu.DM.09G020590.1 | AT5G14420 |
| 57 | A voltage-dependent anion channel (VDAC1) | Soltu.DM.01G009390.1 | AT3G01280 |
| 58 | Encodes a protein with glutamyl-tRNA reductase (GluTR) activity (GLUTR) | Soltu.DM.04G031570.1 | AT1G58290 |
| 59 | Putative AT-hook DNA-binding family protein (AHL17) | Soltu.DM.04G030820.1 | AT5G49700 |
| 60 | A soluble glutamyl-tRNA reductase (GluTR) binding protein (PGR7) | Soltu.DM.03G037680.1 | AT3G21200 |
| 61 | Similar to DEAD/DExH box ATP-dependent RNA helicase (BRR2) | Soltu.DM.06G032390.1 | AT1G20960 |
| 62 | Kunitz family trypsin and protease inhibitor protein | Soltu.DM.03G023510.1 | AT1G73325 |
| 63 | Endonuclease/exonuclease/phosphatase family protein (FRA3) | Soltu.DM.11G003830.1 | AT1G65580 |
| 64 | Zinc finger (C3HC4-type RING finger) family protein (WAV3) | Soltu.DM.04G031560.1 | AT5G49665 |
| 65 | Ni^+^ dependent glyoxalase I homolog ATGLX1(GLX1) | Soltu.DM.02G020230.2 | AT1G11840 |
| 66 | A multiple organellar RNA editing factor, a chloroplast protein (MORF2) | Soltu.DM.06G001960.1 | AT2G33430 |
| 67 | U-box type E3 ubiquitin ligase (PUB32) | Soltu.DM.03G017970.1 | AT3G49060 |
| 68 | Encodes an enolase (ENO2) | Soltu.DM.09G004270.1 | AT2G36530 |
| 69 | U-box type E3 ubiquitin ligase (PUB40) | Soltu.DM.02G032560.1 | AT5G40140 |
| 70 | Cytochrome P450, family 716, subfamily A, polypeptide (CYP716A1) | Soltu.DM.01G015000.1 | AT5G36110 |
| 71 | Nucleolar complex related 3 (NOC3) | Soltu.DM.04G029630.1 | AT1G79150 |
| 72 | U-box type E3 ubiquitin ligase (PUB38) | Soltu.DM.03G027770.1 | AT5G65200 |

**Table S3 Variations in leaf anatomical structure of WT, OE(OE-StPUB40), COE plants under drought stress**

| **Name** | **Treatment** | **Leaf thickness**  **(μm)** | **Palisade tissue thickness**  **(μm)** | **Spongy tissue thickness**  **(μm)** |
| --- | --- | --- | --- | --- |
| WT | CK | 149.75±1.05a | 56.16±0.87a | 65.81±0.76a |
|  | Drought | 136.69±0.83A | 49.72±0.74A | 60.90±0.52A |
| OE-StPUB40 | CK | 145.51±0.58b | 50.90±0.58b | 64.03±0.71ab |
|  | Drought | 132.19±0.72B | 46.70±0.74B | 58.47±0.74B |
| COE | CK | 144.89±0.80b | 49.29±0.30b | 62.60±0.46bc |
|  | Drought | 128.46±0.60C | 42.90±0.59C | 55.99±0.89C |

Data means±SD, n=30. Different small letters indicate statistical differences of leaf structure. Different capital letters indicate statistical differences of leaf structure under drought stress.
