# Supplementary figures and images for: The StUBC18-StPUB40 pair negatively regulate drought stress tolerance and influences tuber yield in potato
Source: Hortic Res. 2025 Jun 10;12(9):uhaf145. doi: 10.1093/hr/uhaf145 (PMC12313339; doi:10.1093/hr/uhaf145)

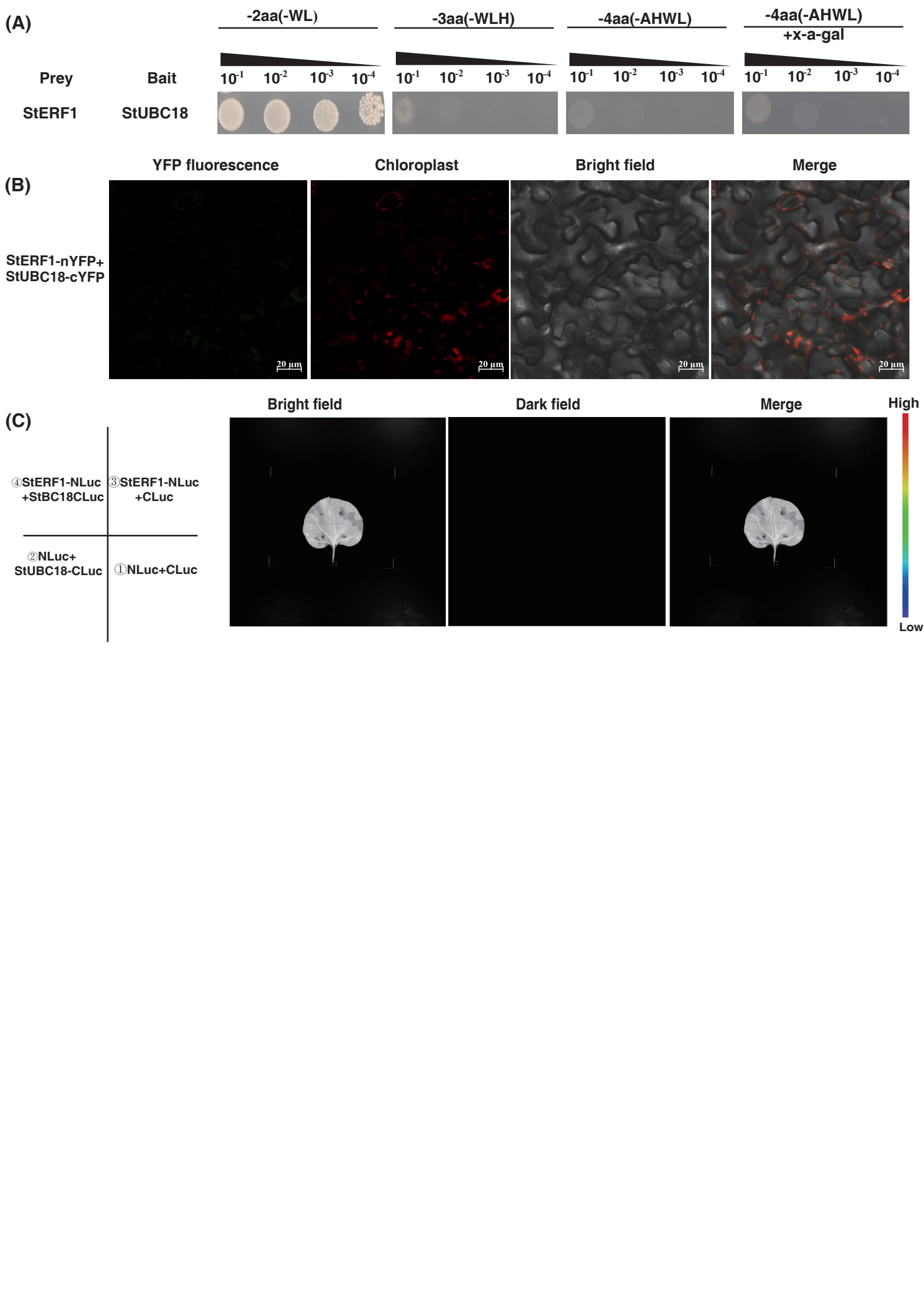

Supplement: Web_Material_uhaf145 [file web_material_uhaf145.zip › FigureS1.pdf]

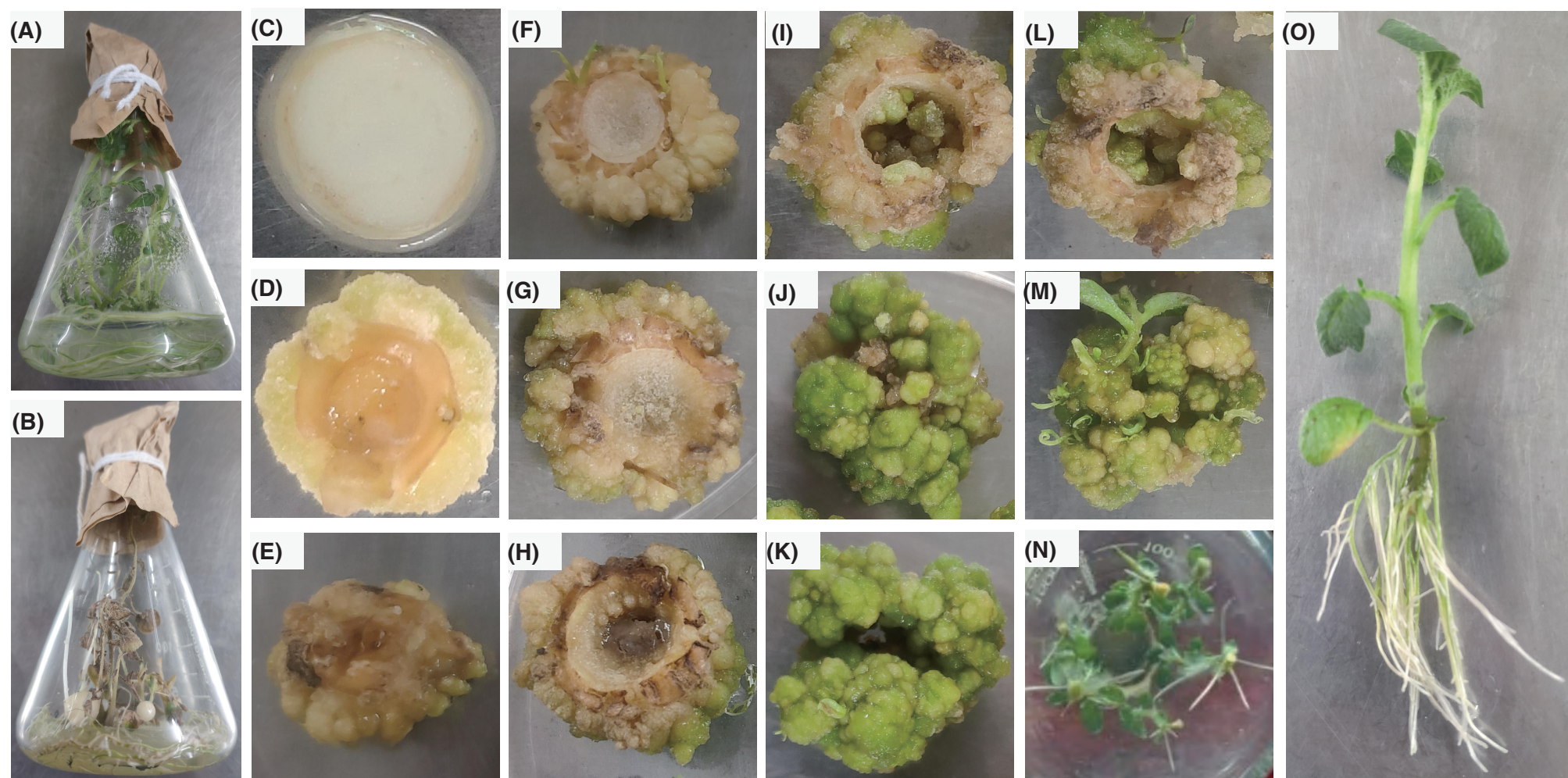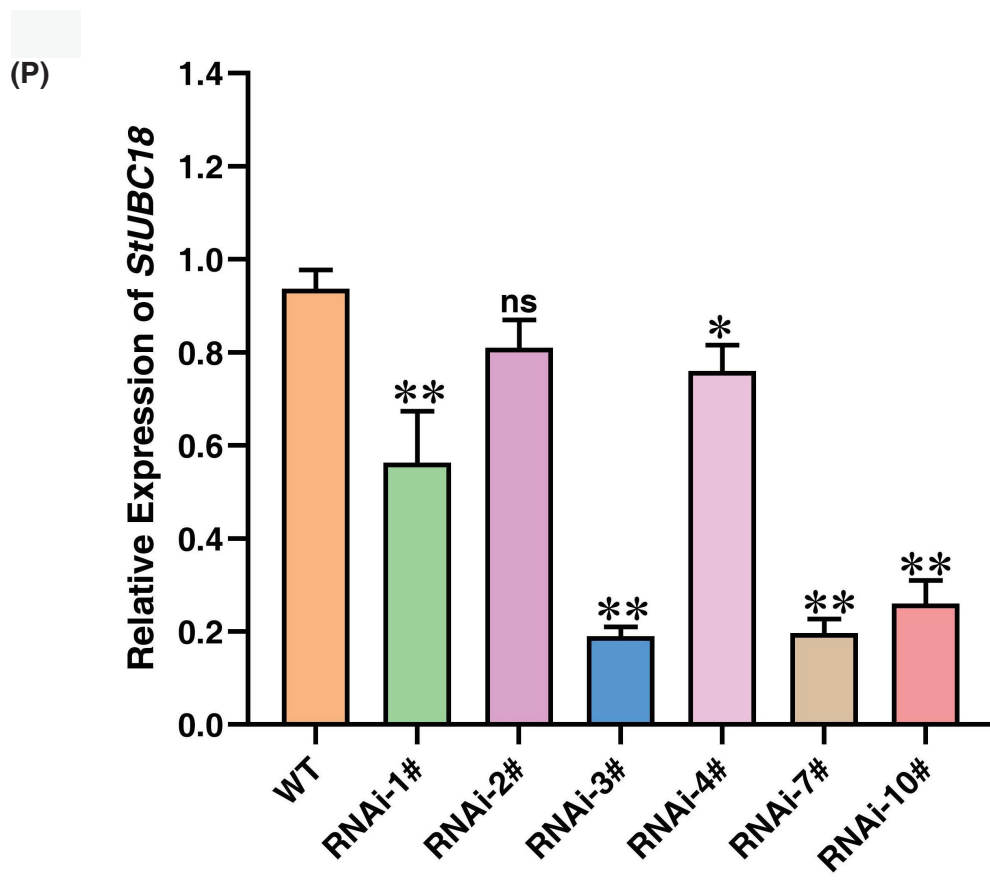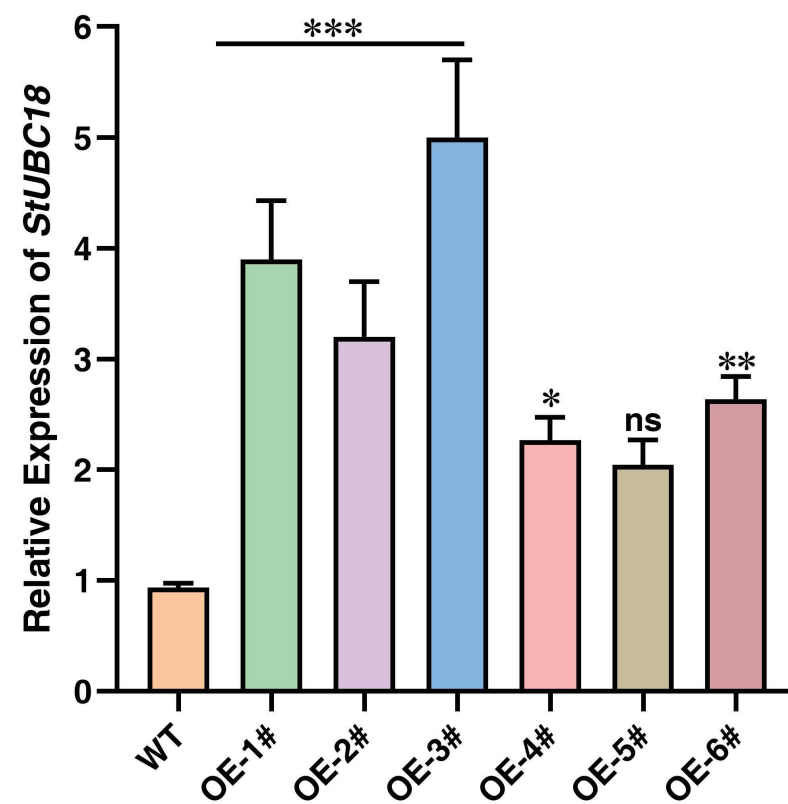

Supplement: Web_Material_uhaf145 [file web_material_uhaf145.zip › FigureS2.pdf]

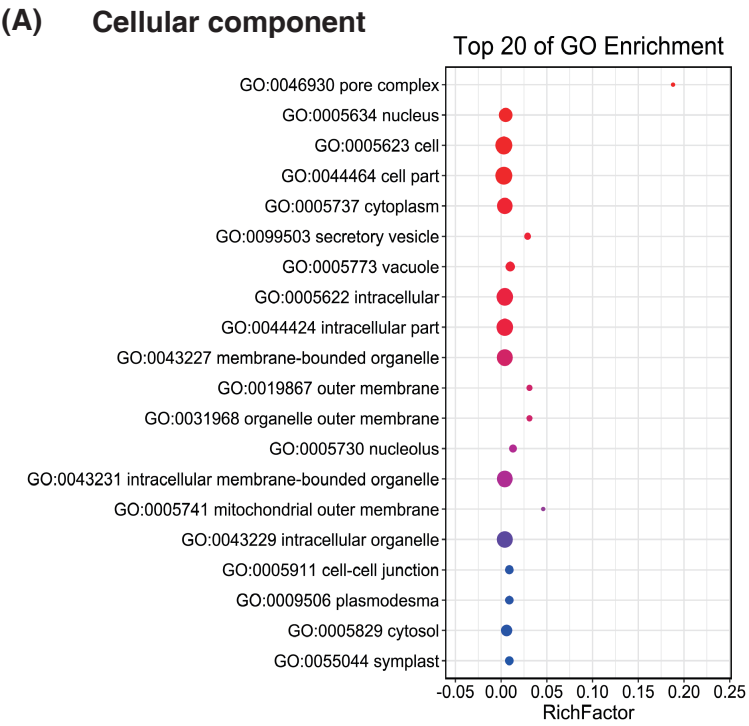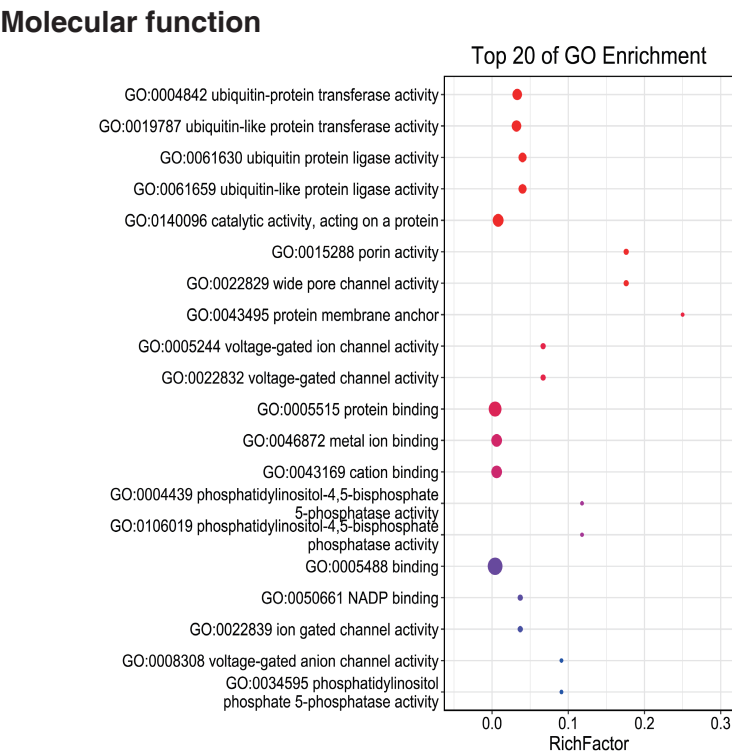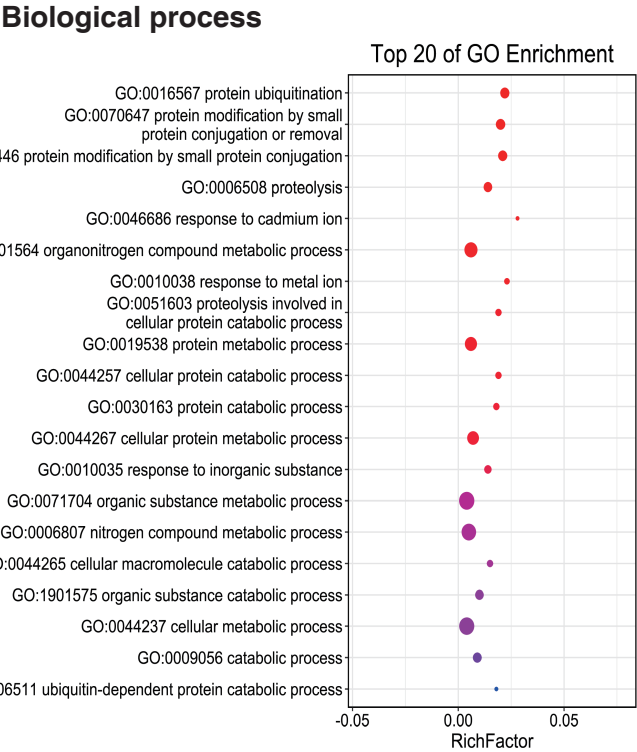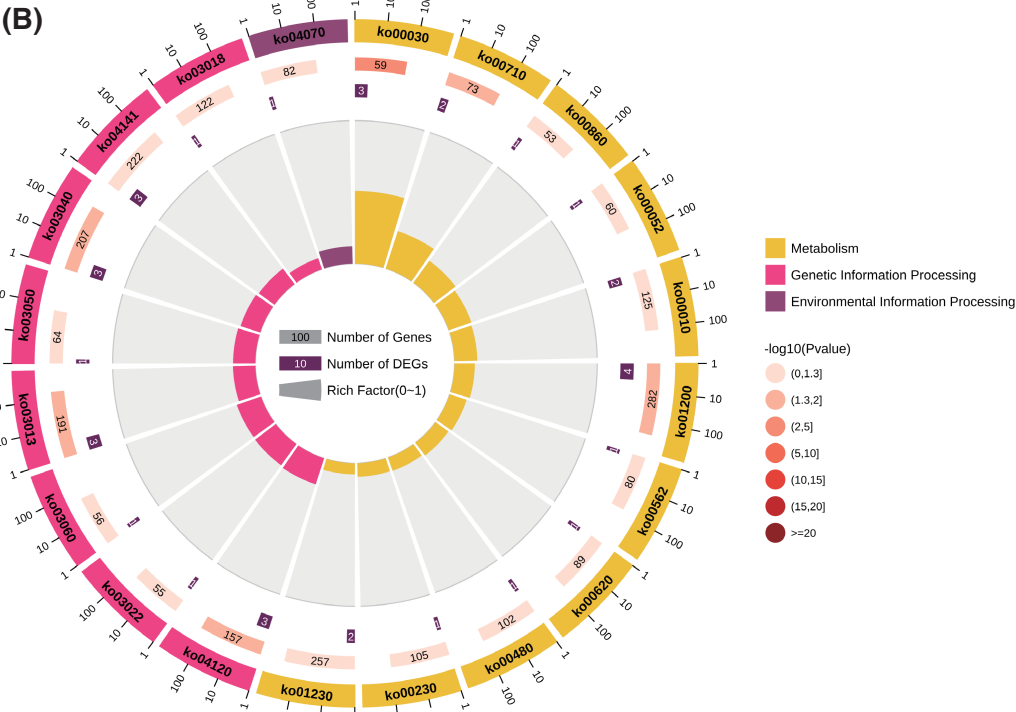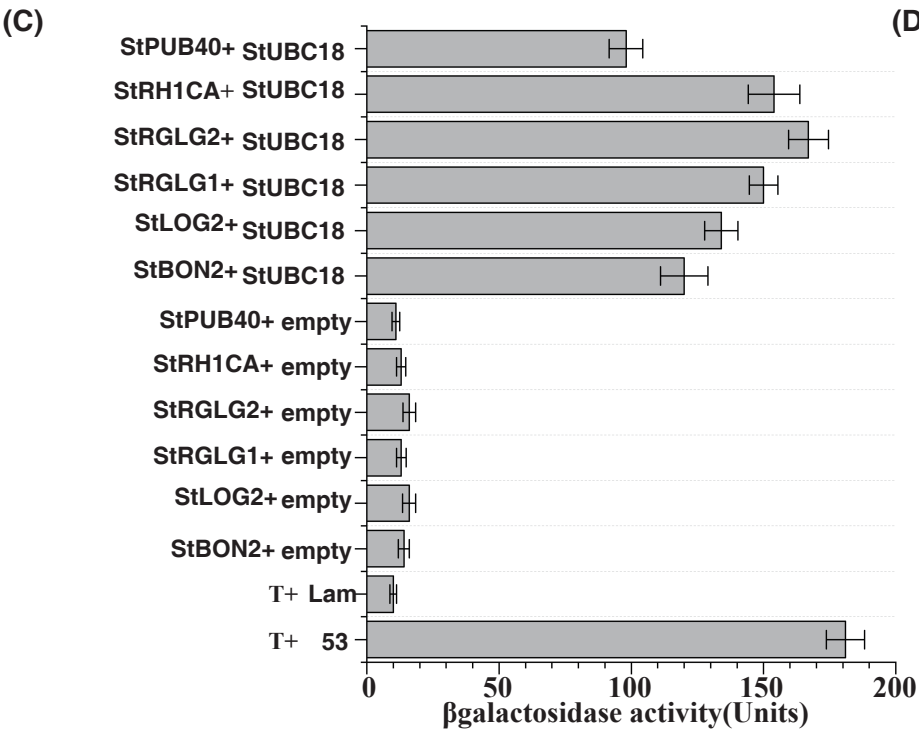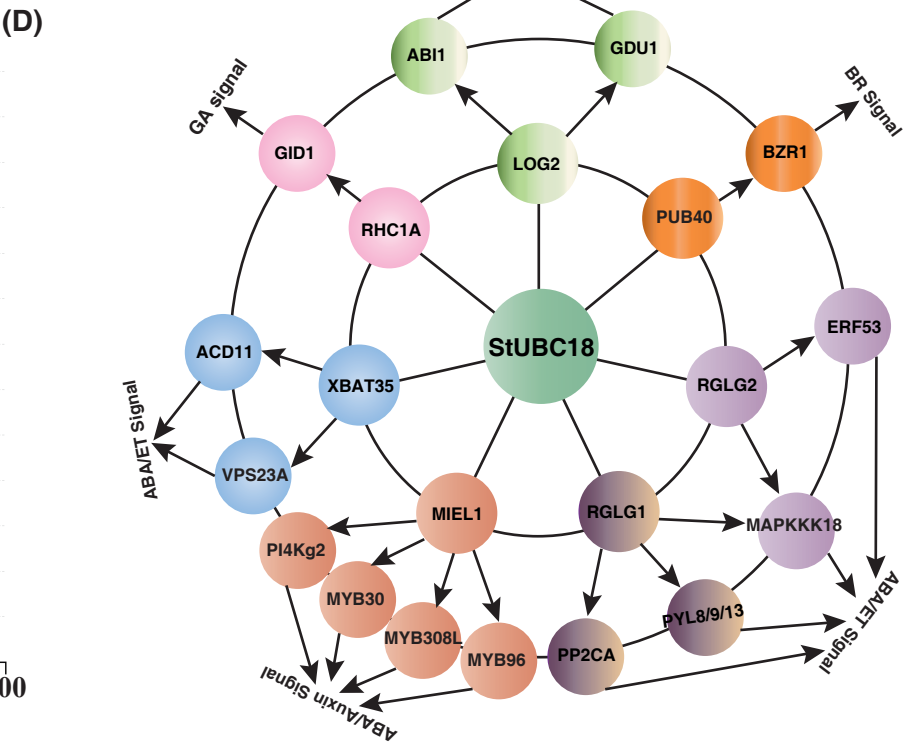

Supplement: Web_Material_uhaf145 [file web_material_uhaf145.zip › FigureS3.pdf]

(A)

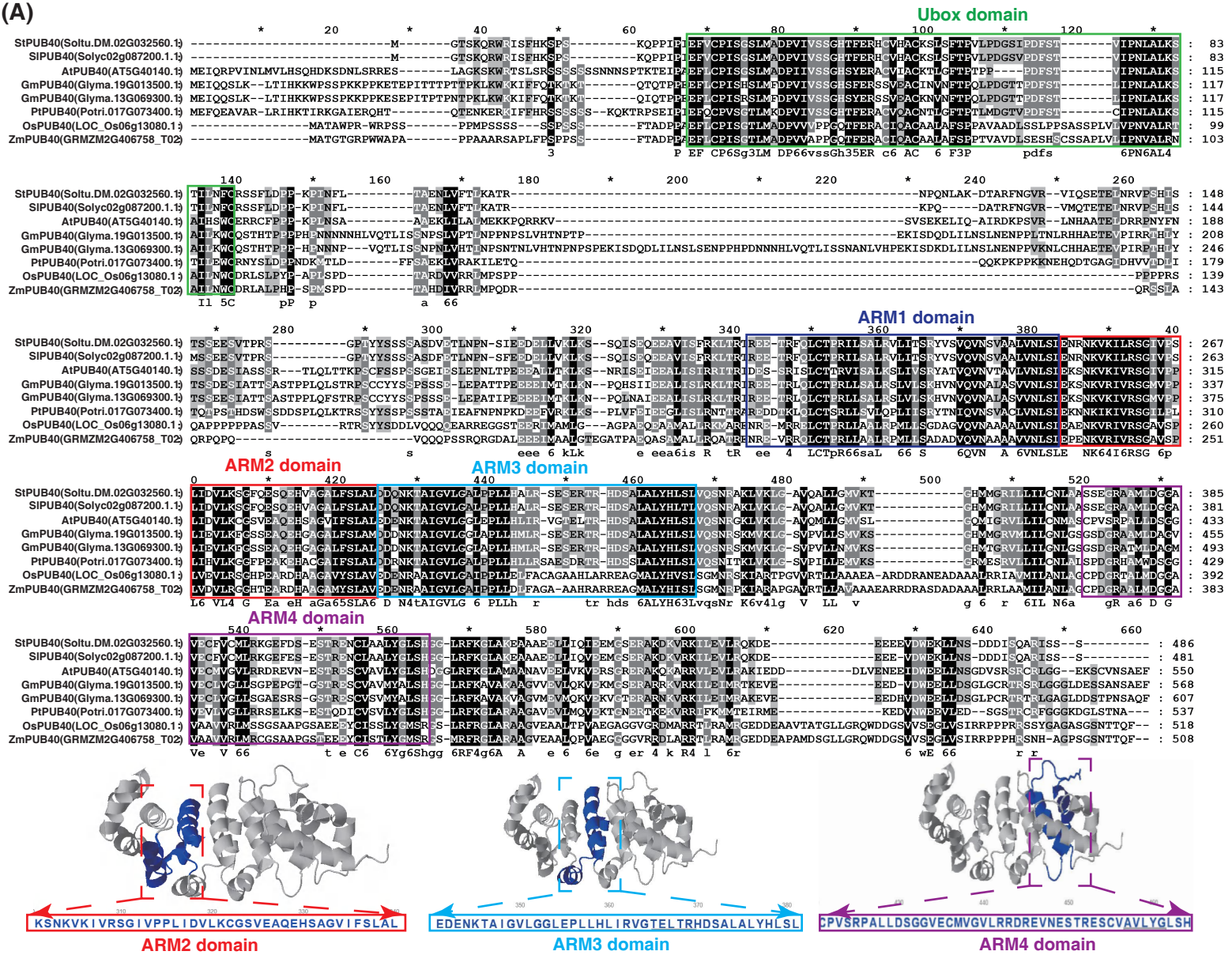

(B)

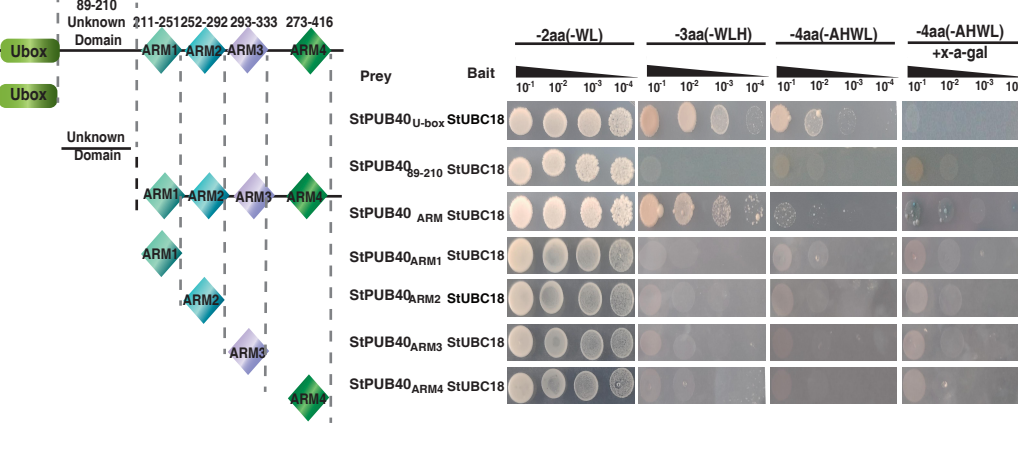

(C)

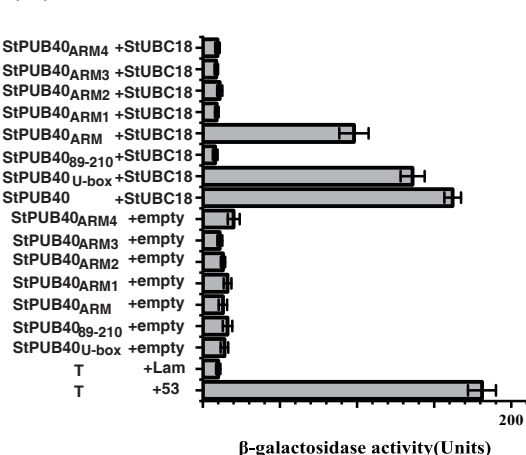

Supplement: Web_Material_uhaf145 [file web_material_uhaf145.zip › FigureS4.pdf]

(A)

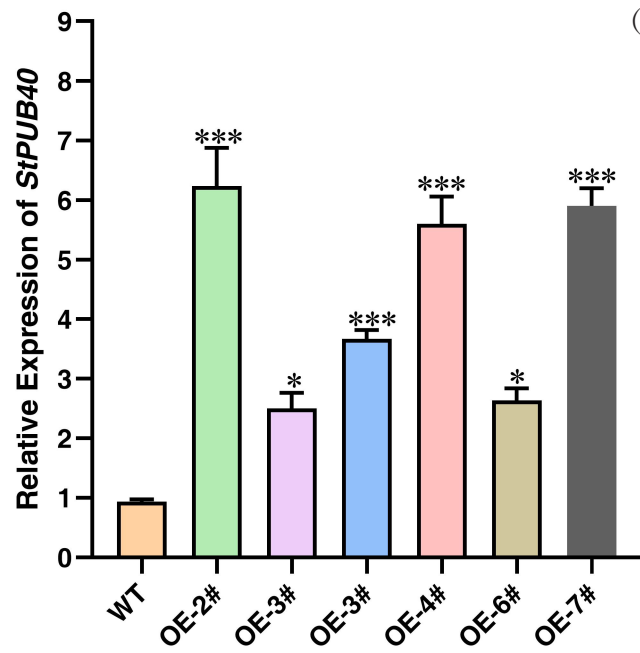

(B)

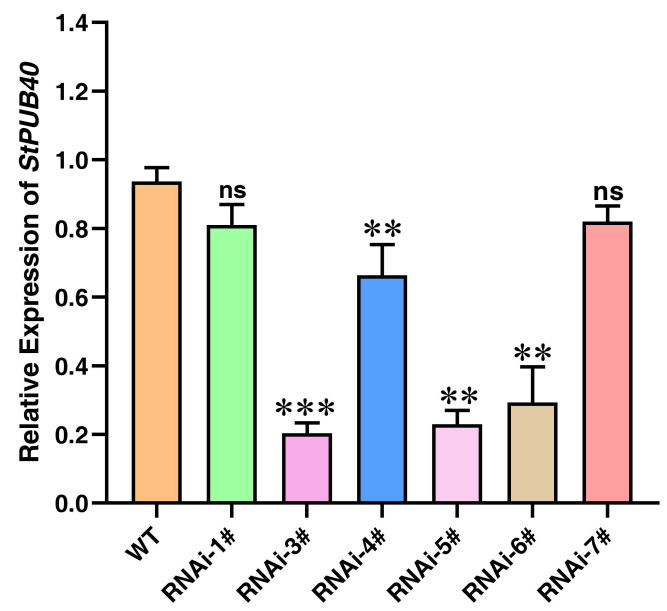

Supplement: Web_Material_uhaf145 [file web_material_uhaf145.zip › FigureS5.pdf]
